# Facile construction of a hyperbranched poly(acrylamide) bearing tetraphenylethene units: a novel fluorescence probe with a highly selective and sensitive response to Zn^2+^†

**DOI:** 10.1039/c7ra13263h

**Published:** 2018-02-05

**Authors:** Xuejing Liu, Yuangong Zhang, Haijing Hao, Wanju Zhang, Libin Bai, Yonggang Wu, Hongchi Zhao, Hailei Zhang, Xinwu Ba

**Affiliations:** College of Chemistry & Environmental Science, Hebei University Baoding Hebei Province 071002 P. R. China haileipoly@126.com zhonggou556@hbu.edu.cn; Hubei Key Laboratory for Processing and Application of Catalytic Materials, Huanggang Normal University Huanggang Hubei Province 438000 P. R. China

## Abstract

Thermo-responsive hyperbranched copoly(bis(*N*,*N*-ethyl acrylamide)/(*N*,*N*-methylene bisacrylamide)) (HPEAM-MBA) was synthesized by using reversible addition–fragmentation chain-transfer polymerization (RAFT). Interestingly, the zinc ion (Zn^2+^) was found to have a crucial influence on the lowest critical solution temperature (LCST) of the thermo-responsive polymer. The tetraphenylethylene (TPE) unit was then introduced onto the backbone of the as-prepared thermo-responsive polymer, which endows a Zn^2+^-responsive “turn-off” effect on the fluorescence properties. The TPE-bearing polymer shows a highly specific response over other metal ions and the “turn-off” response can even be tracked as the concentration of Zn^2+^ reduces to 2 × 10^−5^ M. The decrement of fluorescence intensity was linearly dependent on the concentration of Zn^2+^ in the range of 4–18 μmol L^−1^. The flexible, versatile and feasible approach, as well as the excellent detection performance, may generate a new type of Zn^2+^ probe without the tedious synthesis of the moiety bearing Zn^2+^ recognition units.

## Abstract 2

A novel fluorescent HPEAM-TPEAH, possessing a highly selective and sensitive response to Zn^2+^, was synthesized using RAFT.

## Graphical abstract

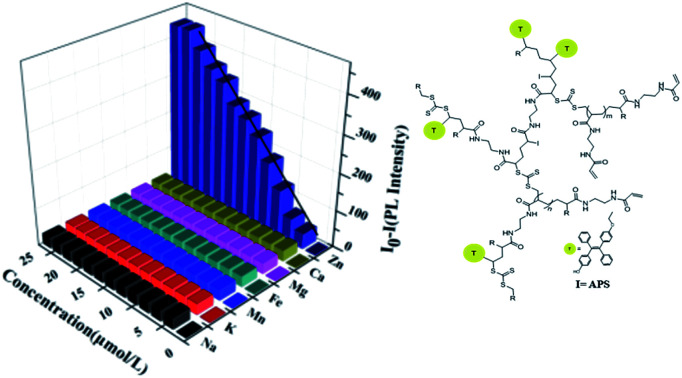

## Introduction

Zinc is widespread in nature, such as in food (vegetables, wheat, fruit, and milk powder), beverages (beer, and wine), animals (cows, and chickens), soil, minerals and the human body. The detection of zinc has raised attention in fields including wastewater treatment, food analysis, environmental monitoring and medical treatment.^1–3^ Zinc content in soil and irrigation water will have an effect on the quality of wheat. Zinc in milk powder has greatly affected the growth of infants. Zinc is one of the important nutritional attributes in many beverages. The zinc ion is the second most abundant transition metal ion in the body and plays an active role in various physiological processes including cell growth, adaptive immunity, gene transcription, apoptosis and neurotransmission.^4–7^ An imbalance in zinc ions is closely associated with diseases, such as diabetes and neurodegenerative diseases.^8–10^ Therefore, the accurate and sensitive detection of zinc ions would be significant for practical application, such as exploring disease mechanisms, food analysis and environmental monitoring. A number of analytical techniques have been employed to detect and quantify zinc in biological systems, including inductively coupled plasma atomic emission spectroscopy, atomic absorption spectrophotometry and radioisotope detection.^11–14^ Comparing with these methods, fluorescent sensor with real-time monitoring, high sensitivity and noninvasive, shows attractive prospect in the detection of intracellular ion levels.^15–17^ The past two decades have witnessed the development of a series of small molecule-based fluorescent zinc ion probes. These synthetic Zn^2+^ probes possessing diverse colors, response mechanisms, kinetics, and binding affinities, have proven to be valuable tools.^18–32^ Despite the progress, for selectively detecting the zinc ions in live cells, a suitable group being of recognition in zinc ion has to be contained in the fluorescent probes. Thus, there remains much to be done to develop novel probes, or seek other way for circumventing this problem.

Since zinc ion is of 3d^10^ electron configuration, it is prone to chelate with other elements (N, S, O) and thereby inducing changes in the fluorescence properties. Zinc ion can also bind with the polymers and result in the conformational changes or cross-linking structures. Zuckermann *et al.*^33^ introduced a high-affinity zinc-binding function on a peptoid (*N*-substituted glycine polymer) two-helix bundle, where the conformational change in polymers was systematically investigated. Yano *et al.*^34^ reported a Zn^2+^ salt of ethylenemethacrylic acid ionomer which possesses pressure responsiveness. The response was caused by the change of coordination structure around zinc ion. The conformational change may also be able to trigger a fluorescence change, and thereby endow a “turn on” or “turn off” response as the polymers binding with the Zn^2+^ in fluorescence polymers.

In this work, we synthesized a backbone temperature-sensitive hyperbranched polyamide in which the temperature sensitivity is imparted by temperature-responsive backbone.^35^ The conformation of polymer chains will be transformed from the extended state into aggregation as the temperature exceeding lower critical solution temperature (LCST). More importantly, we found that the LCST *i.e.* conformational change in polymers can also be affected by the ions due to the existence of N element. Fortunately, in comparison with other cations, Zn^2+^ exhibited a critical influence on LCST. Aggregation-induced emission (AIE) fluorogens firstly presented by Benzhong Tang^36–39^ have attracted considerable interest in the fluorescence probes field for its unique fluorescence emission mechanism. The AIE fluorescent probes can be conferred with “responsive or recognition” feature by introducing a special group that affects the molecular aggregation after reacting with other compound. Taking advantage of AIE feature, Benzhong Tang's group^40^ synthesized a new probe (DEVD-TPE) that might monitor the cell apoptosis. Bo Tang's group^41^ prepared a rapid-response fluorescent probe for hydrogen peroxide. Herein, the introduction of tetraphenylethene into the backbone of the thermo-responsive poly(acryl amide) may create a response of Zn^2+^ to the fluorescence change. A hyperbranched polymer bearing tetraphenylethene units was synthesized *via* RAFT method. The zinc ion stimulates the conformational change of polymer chains and thereby prohibit the intramolecular rotations of AIE units. The TPE-bearing polymer can monitor the zinc ion *via* the decrease in fluorescence intensity, which can be called as a “turn off” response. The development of the AIE-based fluorescent probe for detection of zinc ion without resorting to traditional Zn^2+^ recognition units. The as-prepared polymer can be used as an efficient fluorescent probe for detecting the Zn^2+^ on trace levels.

## Experimental

### Materials

*N*,*N*-dimethylformamide (DMF), ammonium persulfate (APS), *N*,*N*′-methylene bisacrylamide (MBA) and acetone (95%, Tianjin kemiou chemical reagent Co., Tianjin, China) were purified before use. Deuterium Oxide, and Methanol-*d*_4_ were purchased from Sinopharm Chemical Reagent Co., Ltd (Shanghai, China). All the salts were purchased from Tianjin Fuchen Chemical Reagents Factory and used as received. *N*,*N*′-ethyl bisacrylamide (EBA), and *S*,*S*′-bis(*a*,*a*-dimethyl-*a*′′-acetic acid)-trithiocarbonate (BDAAT) were synthesized following the literature procedure and used as chain transfer agent (CTA) in RAFT polymerization.^42^ Tetraphenylethene with one allyloxy group and one hydroxy groups (4-(2-(4-(allyloxy)phenyl)-1,2-diphenylvinyl)phenol, TPEAH) was synthesized following the literature procedure and the ^1^H NMR and MS spectra are given in ESI.†^43^ Deionized water was double-distilled before use.

### Synthesis hyperbranched copoly(bis(*N*,*N*-ethyl acryl amide)/(*N*,*N*-methylene bisacrylamide)) (HPEAM-MBA)

**Scheme 1:**
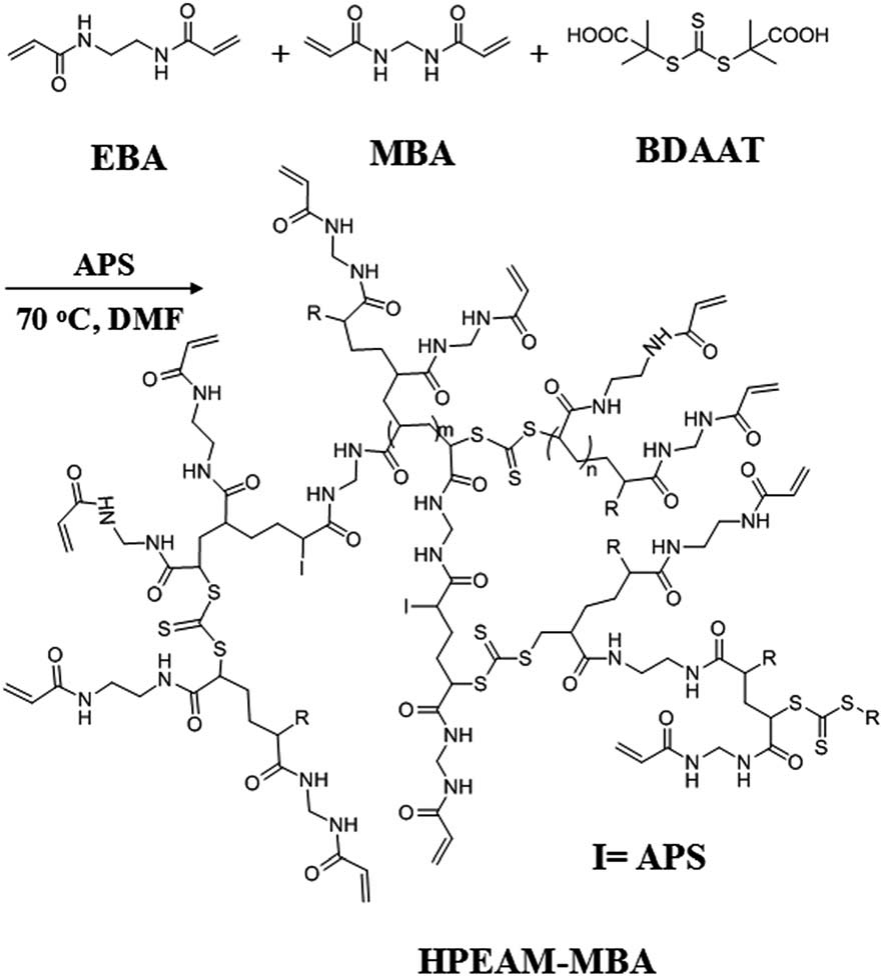
Synthesis of HPEAM-MBA.

Polymerization was carried out in a glass tube that sealed with a rubber cap under nitrogen atmosphere. EBA (336 mg, 2 mmol), MBA (46.2 mg, 0.3 mmol), BDAAT (32.43 mg, 0.115 mmol), APS (52.44 mg, 0.23 mmol) and DMF (8 mL) were added to a Schlenk tube. Oxygen was removed by repeated vacuum-nitrogen cycles. Then, the polymerization was conducted at 70 °C in an oil bath for 24 h. Afterwards, the obtained polymer was precipitated by dropping the solution into a large excess of acetone to remove the excessive monomers and impurities. The precipitated polymer was separated by centrifugation, and then dissolved in water. The final product HPEAM-MBA was given after lyophilization (Scheme 1).

### Synthesis hyperbranched copoly(bis(*N*,*N*-ethyl acryl amide)/4-(2-(4-(allyloxy)phenyl)-1,2-diphenylvinyl)phenol) (HPEAM-TPEAH)

**Scheme 2:**
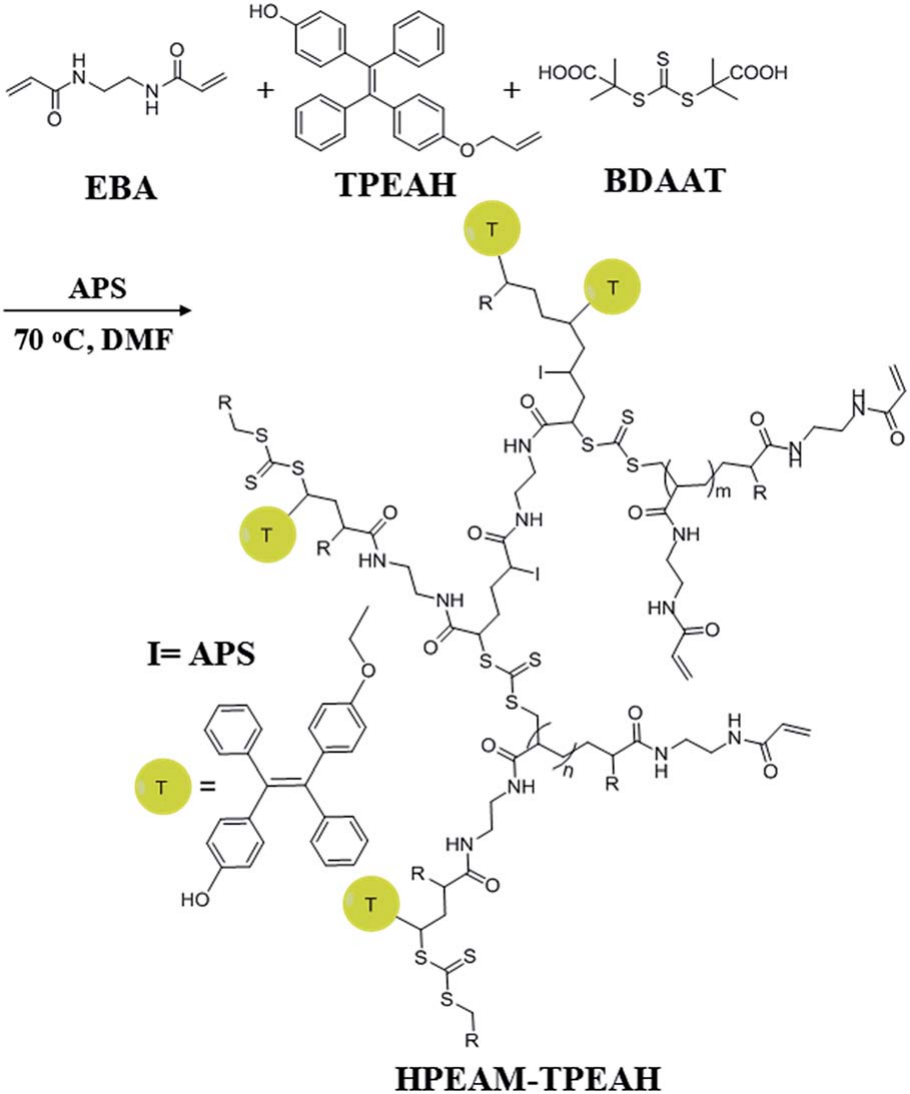
Synthesis of HPEAM-TPEAH.

EBA (336 mg, 2 mmol), MBA (46.2 mg, 0.3 mmol), BDAAT (32.43 mg, 0.115 mmol), APS (52.44 mg, 0.23 mmol), TPEAH (5 mmol, 20 mg) and DMF (8 mL) were added to a Schlenk tube. Oxygen was removed by repeated vacuum-nitrogen cycles. Then, the polymerization was conducted at 70 °C in an oil bath for 24 h. Afterwards, the obtained polymer was precipitated by dropping the solution into a large excess of acetone to remove the excessive monomers and impurities. The precipitated polymer was separated by centrifugation, and then dissolved in water. The final product HPEAM-TPEAH was given after lyophilization (Scheme 2).

### Measurement of lower critical solution temperature (LCST)

LCSTs were defined as the temperature corresponding to 50% transmittance of aqueous solution during the heating process. UV-visible 2550 Spectrophotometer (Shimadzu, Japan) at 500 nm with the heating rate as 0.1 °C min^−1^, and the UV-vis spectra of polymer solution was also obtained through same spectrophotometer from 250 nm to 400 nm. Equivalent amounts of polymers were added to 4.0 mL of aqueous solution of different salts. The mixture was cooled at 4 °C for 10 min to ensure that the polymer was dissolved completely.

### Cytotoxicity study

The cytotoxicity of HPEAM-TPEAH on Hela cells was examined as follows: Hela cells (5 × 103 cells) in 100 μL of DMEM containing 10% FCS were plated in a 96-well plate and incubated for 24 h in a humidified atmosphere of 5% CO_2_ in air at 37 °C (Sanyo, Model MCO-18AIC, Japan) Then 100 μL of a HPEAM-TPEAH in DMEM containing 10% FCS and 2% DMSO was added to each well. Incubation was carried out for 24 h at a HPEAM-TPEAH concentration of 0.5 μM (final DMSO content was 1% in all cases). Cells without HPEAM-TPEAH treatment were used as control group. After 24 h of treatment, MTT dye solution (20 μL, 5 mg mL^−1^) was added to each cell. Cells were incubated for another 4 h and then analyzed using a microplate spectrophotometer (BioRad Model 3550, USA) at 570 nm. The percentage cell survival was calculated by normalization with respect to the value for no HPEAM-TPEAH treatment.

### Characterization

Infrared spectroscopy was measured on a Varian 640-IR Fourier infrared spectrometer (Varian, Palo Alto, CA, USA) by using KBr pellets and solution in D_2_O. CaF_2_ transmission windows and 0.05 mm Mylar spacers were used. For each spectrum, 256 interferograms of 2 cm^−1^ resolution were co-added. From the spectrum of each sample, a corresponding D_2_O spectrum was subtracted. All the spectra were baseline-corrected and normalized. All data processing was performed with Resolution software. The samples were investigated at mass concentration of 0.5 wt% in D_2_O. ^1^H NMR spectra were recorded on a Bruker AVANCE-600 600 MHz spectrometer (Bruker, Germany). The chemical shifts are given in parts per million (ppm). Fluorescence spectrum analysis was measured on the RF-5301-PC (Shimadzu, Japan) fluorescence spectrometer light-transmittance of the polymer solution was measured on a temperature-controlled. Molecular weight, polydispersity, Mark–Houwink index (*α*) and intrinsic viscosity (IV) were determined by Viscotek 270-MDSEC (Malvern Instruments Ltd., Malvern, US) equipped with the differential refractive index (RI), viscometer, and two-angle light scattering (LS, Malvern Instruments Ltd., Malvern, US) triplet detectors. The eluent was 0.1 mol L^−1^ NaNO_3_ aqueous solution at a flow rate of 1 mL min^−1^ at 15 °C. For MDSEC, narrow dispersion polyethylene oxide std-PE022K was used to calibrate the instrument. TEM studies of copolymers were performed on a Tecnai G2 F20 S-TWIN (FEI Company, Hillsboro, USA) dedicated to cryogenic temperatures, operated at an acceleration voltage of 200 kV at low electron-dose conditions. A small drop of clear and cloudy copolymers solution (1 mg mL^−1^) containing 1 wt% phosphotungstic acid (PTA) was placed on the Cu grid with holey carbon film held by tweezers of the vitrification system Vitrobot (FEI Company, Hillsboro, USA). DLS measurements were performed on a Brookhaven BI-200 goniometer (Brookhaven, New York, USA) with vertically polarized incident light of wavelength *λ* = 532 nm supplied by a helium–neon laser operated at 75 mW and a Brookhaven BI-4700 AT digital autocorrelator (Brookhaven, New York, USA).

## Results and discussions

In a previous study, hyperbranched poly(bis(*N*,*N*-propyl acryl amide))^44^ and poly(bis(*N*,*N*-ethyl acrylamide))s^45^ had been successfully synthesized with high yield by reversible addition–fragmentation chain transfer polymerization (RAFT) method. In addition, the lower critical solution temperature (LCST) often ranges from 19 to 28 °C, and 6 to 17 °C, respectively. In this study, the synthesis hyperbranched copoly(bis(*N*,*N*-ethyl acryl amide)/(*N*,*N*-methylene bisacrylamide)) (HPEAM-MBA) was conducted in order to achieve a desirable LCST near human body temperature. Fortunately, the LCST of HPEAM-MBA is determined to be 36.4 °C (details were shown in the ESI†).

It is known that the monomer possessing a multi vinyl monomer (MVM) is usually employed to fabricate polymers with crosslinked network structures, even if only small amounts of MVM participates in the free radical polymerization. However, Sherrington^46^ and Guan^47^ respectively prepared hyperbranched polymers with MVM as branching units *via* a chain transfer controlled free radical polymerization. The crosslinking was effectively inhibited by using thiol or catalytic chain transfer catalyst species. In Sato's study,^48–51^ the MVM was also used as the branching species in which the radical polymerization was well controlled by a chain termination. Recently, controlled free radical polymerization was used in the MVM reaction system, which is able to reduce the reaction rate and avoid cross-linking in polymerization. In addition, the preparation of hyperbranched polymers can also be well controlled by copolymerization with other monomers *via* living polymerization. For an example, Wang and Howdle^52^ synthesized hyperbranched polymers with highly branched structures *via* atom transfer radical polymerization (ATRP). In our study, RAFT polymerization was adopted for the synthesis of hyperbranched copoly(bis(*N*,*N*-ethyl acryl amide)/(*N*,*N*-methylene bisacrylamide)) (HPEAM-MBA). As shown in Scheme 1, the RAFT polymerization of MVM consists of three stages: initiating, propagation and terminal stage. The feed ratio of [monomer] : [CTA] reaches 20 : 1 in system. The addition of BDAAT effectively reduced the rate of the free radical polymerization and avoided the crosslinking reaction. The polymerization was carried out in *N*,*N*-dimethyl formamide (DMF) for 24 h, the resulting polymers were obtained by a precipitation method in acetone. HPEAM-MBA, a polymer with LCST = 36.4 °C, *M*_w_ = 4.33 × 10^4^ (*M*_w_/*M*_n_ = 1.22), was well characterized by FTIR, ^1^H NMR and ^13^C NMR, and the details were shown in the ESI,† respectively. The Mark–Houwink index (*α*) was calculated as 0.256, matches well with the inherent character of hyperbranched polymers.^53,54^

**Fig. 1:**
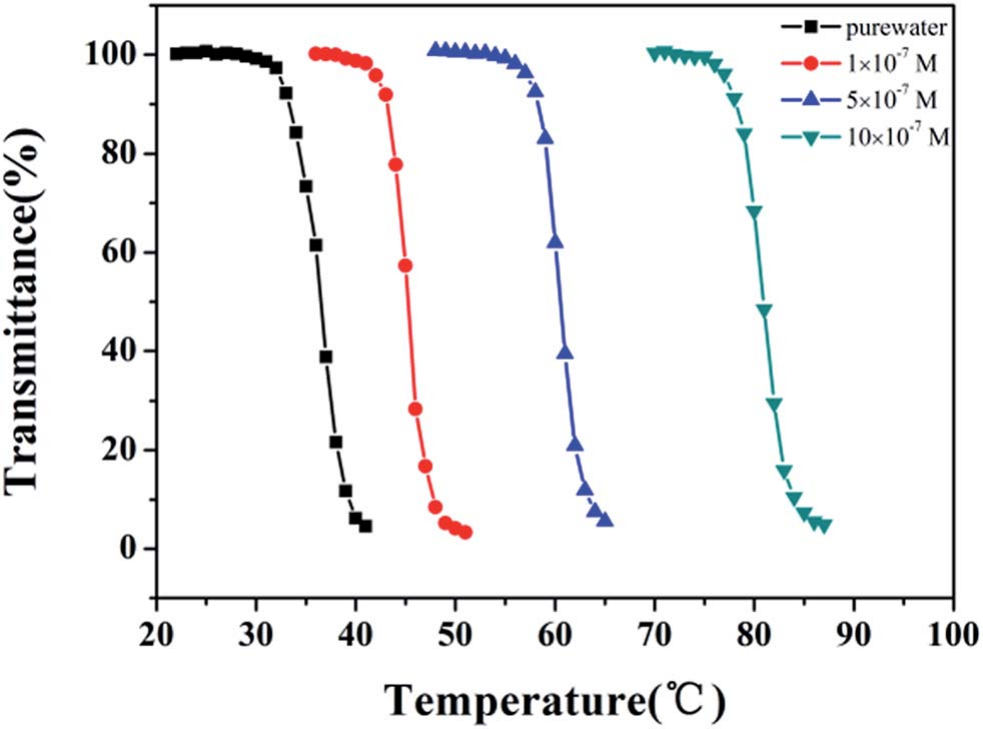
The curves of transmittance *versus* temperature of HPEAM-MBA solution containing Zn^2+^. Polymer concentration: 5 mg mL^−1^; heating rate: 0.1 °C min^−1^.

**Fig. 2:**
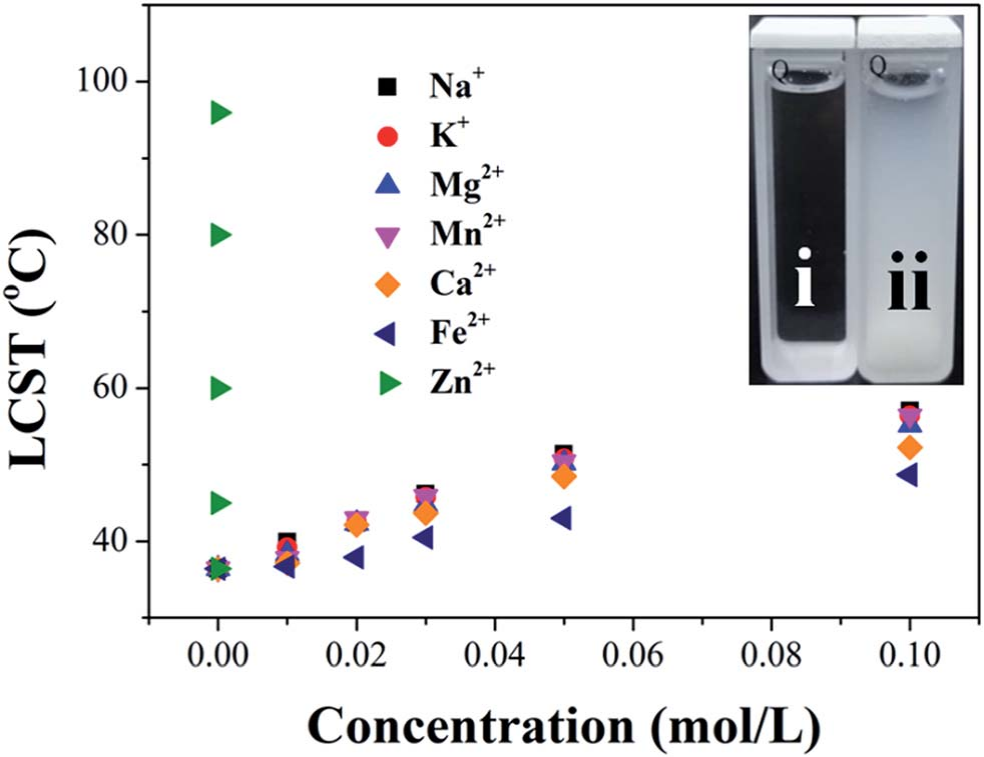
Influence of different cations (Na^+^, K^+^, Mg^2+^, Ca^2+^, Mn^2+^, Fe^2+^ and Zn^2+^) on the LCST of HPEAM-MBA ((i) shows the HPEAM-MBA solution when the temperature is lower than LCST; (ii) shows the HPEAM-MBA solution when the temperature is higher than LCST).

The nitrogen in the backbone of as-prepared polymer is able to coordinate with metal ion to form M^*n*+^–N_*x*_ complex and result in the changes in hydration. Therefore, the LCST of HPEAM-MBAs may also be changed by the addition of metal ions. The influence of metal ions, including Na^+^, K^+^, Mg^2+^, Ca^2+^, Mn^2+^, Fe^2+^, Zn^2+^, on the LCST was investigated. Zn^2+^ was selected as a represent metal ion whose turbidity curves that reflect the influence of its concentration on the LCST values are shown in Fig. 1. As expected, the turbidity curves are shifted to high temperature with the concentration of Zn^2+^ increasing. The turbidity curves of other metal ions are displayed in the ESI† respectively. After extracted the LCST values, the plot of LCST values against concentration are illustrated in Fig. 2. It can be observed from the Fig. 2 that all of these metal ions exhibit the hydrotropy ability which can be owing to the salting-in effect. The sequence salting-in effect is Fe^2+^ < Ca^2+^ < Mg^2+^ < Mn^2+^ < K^+^ ≈ Na^+^ ≪ Zn^2+^, which is consistent with the typical Hofmeister cation series.^55–57^ It should be noted that the tendency of the plots of LCST *vs.* [Zn^2+^] displays a significant difference over than those of other metal ions that the LCST is dramatically increased even [Zn^2+^] < 1 × 10^−5^ M.

**Fig. 3:**
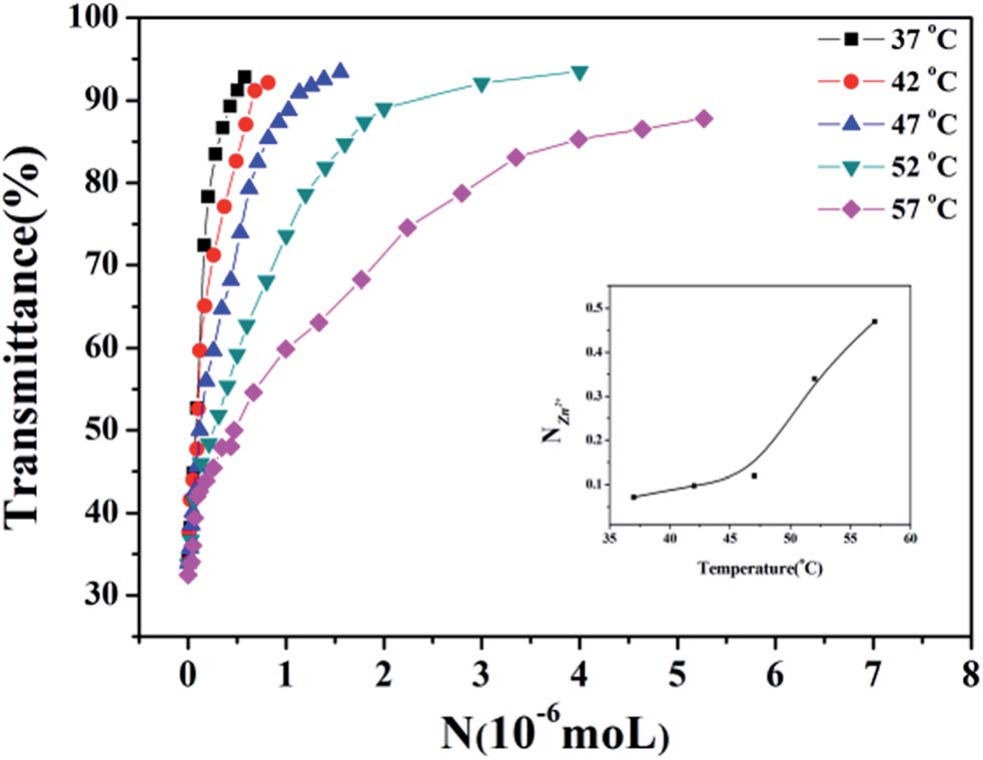
The curves of transmittance% *vs. N*_Zn^2+^_ of HPEAM-MBA at different temperatures. Polymer concentration: 5 mg mL^−1^.

**Fig. 4:**
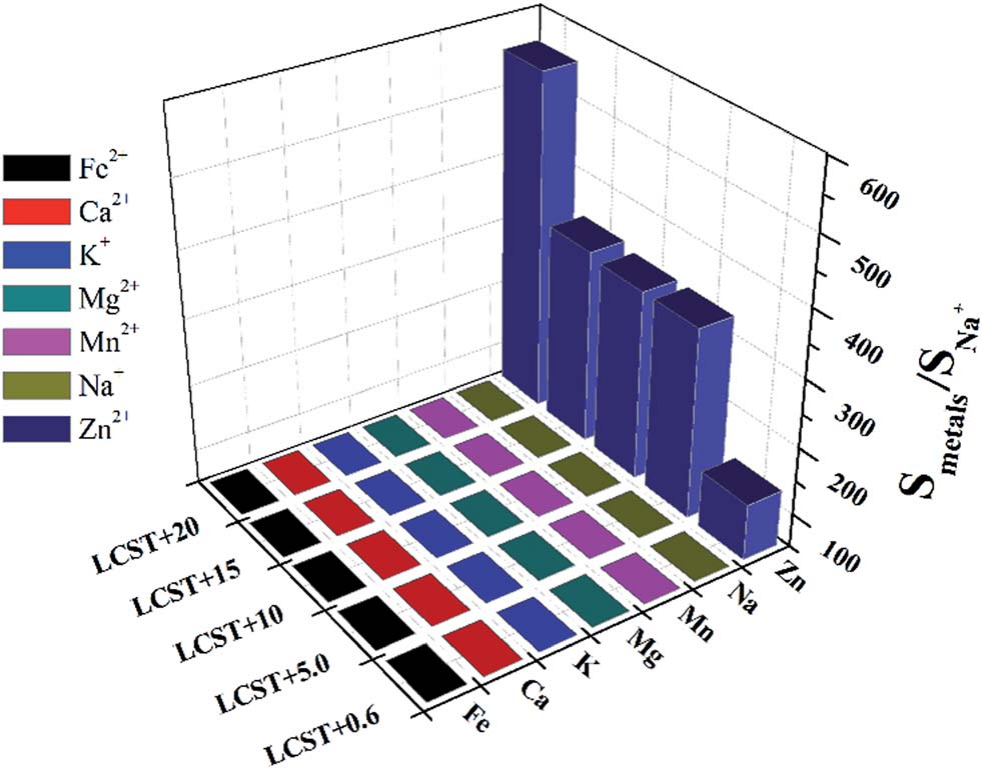
The three-dimensional histogram of temperature *vs. S*_metals_/*S*_Na^+^_.

In order to compare the influence of Zn^2+^ on the LCST with other metal ions more quantitatively, the parameter *S* (named sensitivity) is introduced and defined as the reciprocal of *N* when the transmittance% of the solution is 50%. For getting the parameter *S*, the transmittance% of the solution under different concentration of metal ions was recorded and shown as Fig. 3. All of the curves display a similar tendency that the transmittance% of the solution increases as the *N*_Zn^2+^_ ascends. Also, the temperature affects the amount of the Zn^2+^. Accompany with temperature of the solution rising up, the *N*_Zn^2+^_ obviously increase. As pointed out in the aforementioned part, high temperature will destroy the hydrogen bonds between the water molecules and amide groups, which means the hydrophobicity of HPEAM-MBAs is proportional to the temperature of solution, therefore the more *N*_Zn^2+^_ is needed as the temperature getting higher. The curve of *N*_Zn^2+^_ with temperature is inserted into the lower right corner of Fig. 3. According with the definition of *S*, the *N*_Zn^2+^_ decreases as the increase of *S*. The curves of transmittance% *vs. N* of other metals are in the ESI† and display the similar tendency to that in Fig. 3. Although the value of *S* is the highest as the temperature is closed to LCST, the *S*_Zn^2+^_/*S*_metals_ is not the highest value which reveals that the HPEAM-MBAs solution does not display good selectivity towards Zn^2+^. Encouragingly, the value of the *N*_Zn^2+^_ is far below the *N* of other metals when the temperature of solution is 20 degrees above the LCST, and the ratio of *S*_Zn^2+^_/*S*_metals_ can reach 1.28 × 10^3^. To graphically show the selectivity of HPEAM-MBAs solution towards Zn^2+^, the *S*_Na^+^_ is chose as the reference because that the Na^+^ is the most abundant ion in the human body. As shown in Fig. 4, the ratio of *S*_Zn^2+^_/*S*_Na^+^_ is 5.75 × 10^2^ when the temperature is 57 °C. These results indicate that HPEAM-MBAs solution is of high sensitivity in assessing Zn^2+^, and the HPEAM-MBAs solution can selectively recognize the Zn^2+^ when the environment temperature is 20 degrees above the LCST.

**Fig. 5:**
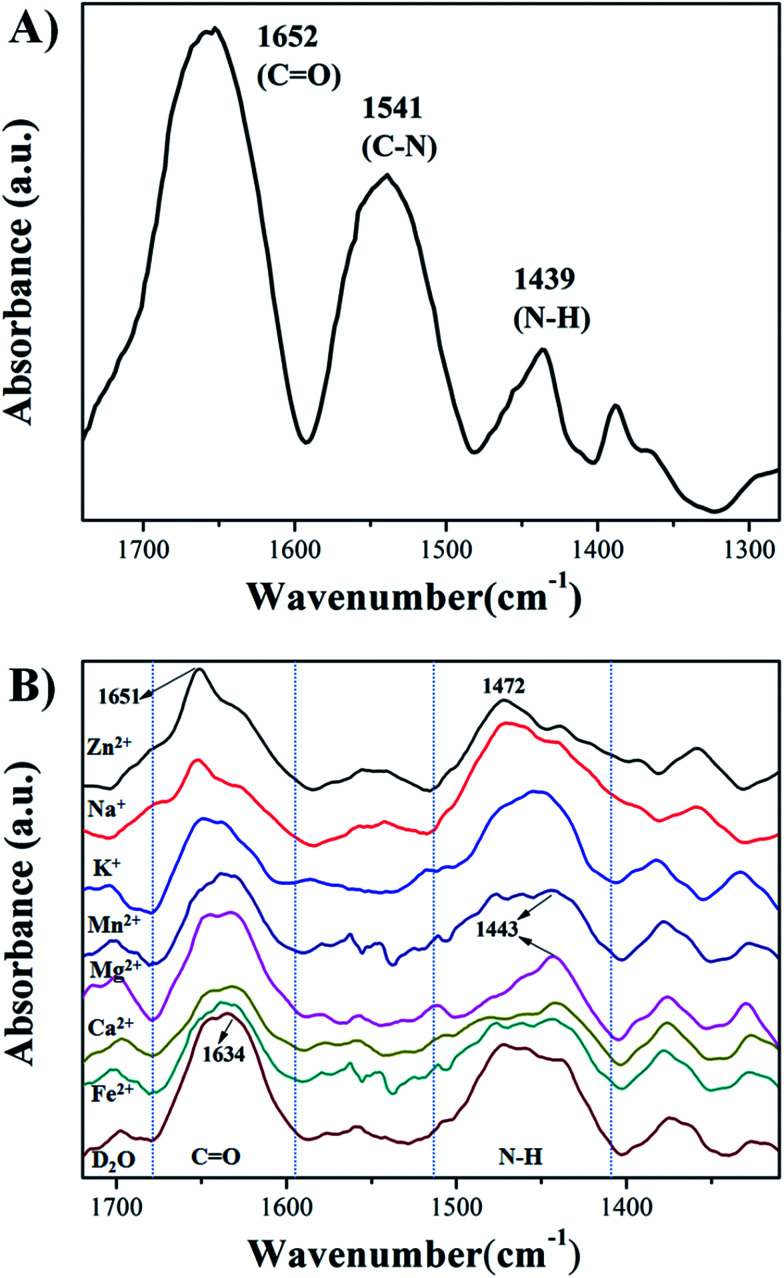
Infrared absorption spectra of HPEAM-MBA ((A) shows the infrared absorption spectrum of HPEAM-MBA measured in KBr; (B) shows the infrared absorption spectrum of HPEAM-MBA measured in D_2_O with or without the metal ions, respectively).

Infrared absorption spectra was employed to investigate the interaction of HPEAM-MBA and Zn^2+^. Fig. 5(A) shows the infrared absorption spectrum of HPEAM-MBA (in KBr). The characteristic adsorptions at 1652 cm^−1^ represents the stretching vibration of C=O, the peak located at 1541 cm^−1^ corresponds to C–N stretching vibration in the *trans*-associated secondary amide, and the signal at 1439 cm^−1^ is the N–H bending vibration in *cis*-amide. Fig. 5(B) is the infrared absorption spectra of HPEAM-MBA measured in D_2_O, and measured with the presence of the metal ions in D_2_O, respectively. Due to the interaction between the D_2_O and HPEAM-MBA, the signals corresponding to C=O and N–H shift to the lower wavenumber region. For Na^+^, K^+^, Mg^2+^ and Ca^2+^, the characteristic adsorptions of N–H shifted to the lower wavenumber region, while the vibrational peak of C=O stretching unchanged, which indicates that the addition of Na^+^, K^+^, Mg^2+^ and Ca^2+^ promotes the dissociation of amide group. As the Zn^2+^, the characteristic adsorptions of C=O and N–H shifted from 1634 cm^−1^ to 1651 cm^−1^ and 1450 cm^−1^ to 1472 cm^−1^ respectively. Evidently, the Zn^2+^ binds with not only N atom, but also the O atom which can be due to its 3d^10^ electron configuration. Therefore, Zn^2+^ shows a much stronger salting-in effect as comparing to those of other metal ions.

Because that the HPEAM-MBA solution shows the feature of selectively recognizing the Zn^2+^ as the environment temperature is 20 degrees higher than the LCST, the HPEAM possessing lower LCST was selected as the platform to copolymerize with TPEAH. The TPE-bearing polymer was synthesized by using reversible addition–fragmentation chain-transfer polymerization (RAFT). It shows a highly specific response over other metal ions. The mechanism of polymerization was shown in Scheme 2 and the detail was shown in ESI.† The resulting copolymer HPEAM-TPEAH(with yield as 57%.) was characterized by FTIR, and ^1^H NMR, and the detailed assignment is shown in ESI.† The ratio of EAM\TPEAH is 2.9 on basis of the ^1^H NMR results.

**Fig. 6:**
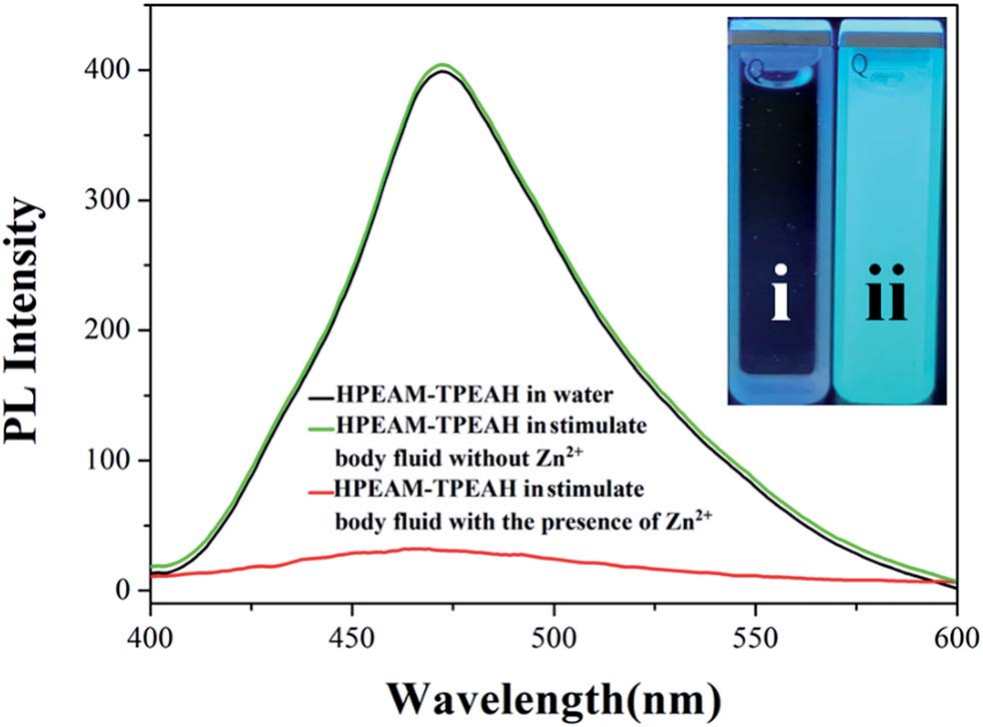
Fluorescence intensity curves of HPEAM-TPEAH under simulate body fluid with (i) or without Zn^2+^ (ii). The simulate body fluid contains 140 mmol L^−1^ Na^+^, 5.5 mmol L^−1^ K^+^, 1.33 mmol L^−1^ Ca^2+^, 5 mmol L^−1^ Mg^2+^, Zn^2+^ and 3.0 mmol L^−1^ Fe^2+^. The concentration of Zn^2+^ is 0.02 mmol L^−1^.

The HPEAM-TPEAH is difficult to be dissolved in water, while the aqueous system containing HPEAM-TPEAH displays an obvious fluorescence emission which can be owing to the aggregation-induced emission (AIE) effect of TPE units. Generally, the incorporation with metal ions would raise the solubility of the polymers in aqueous system and thereby result in the decreasing of fluorescence intensity, which can be named as the “turn off” response. The “turn off” response of HPEAM-TPEAH on the metal ions including Zn^2+^, Mn^2+^, Na^+^, K^+^, Ca^2+^, Mg^2+^ and Fe^2+^ were investigated respectively. The results depicted on ESI† shows that the “turn off” response is hard to be detected for Mn^2+^, Na^+^, K^+^, Ca^2+^, Mg^2+^ and Fe^2+^, while the addition of HPEAM-TPEAH into the Zn^2+^ solution triggers a dramatically decrease in fluorescence intensity. Furthermore, a simulate body fluid (Na: 140 mmol L^−1^; K^+^: 5.5 mmol L^−1^; Ca^2+^: 1.33 mmol L^−1^; Mg^2+^: 5 mmol L^−1^; Fe^2+^: 3.0 mmol L^−1^) was prepared, where the “turn-off” cannot be detected with the addition of HPEAM-TPEAH into simulate body fluid (shown in Fig. 6). While the fluorescence intensity significantly decreased as the addition of Zn^2+^ into the above system. Results of the linear regression for *I*_0_–*I* and [Zn^2+^] show that the linear correlation coefficient (*R*^2^) is greater than 0.99 which indicates that the *I*_0_–*I* and [Zn^2+^] shows a good linearity relationship in the range from 2 × 10^−6^ to 2 × 10^−5^ mol L^−1^ (details were shown in ESI†). The results show that HPEAM-TPEAH exhibits a highly specific “turn-off” response of Zn^2+^ over other metal ions.

**Fig. 7:**
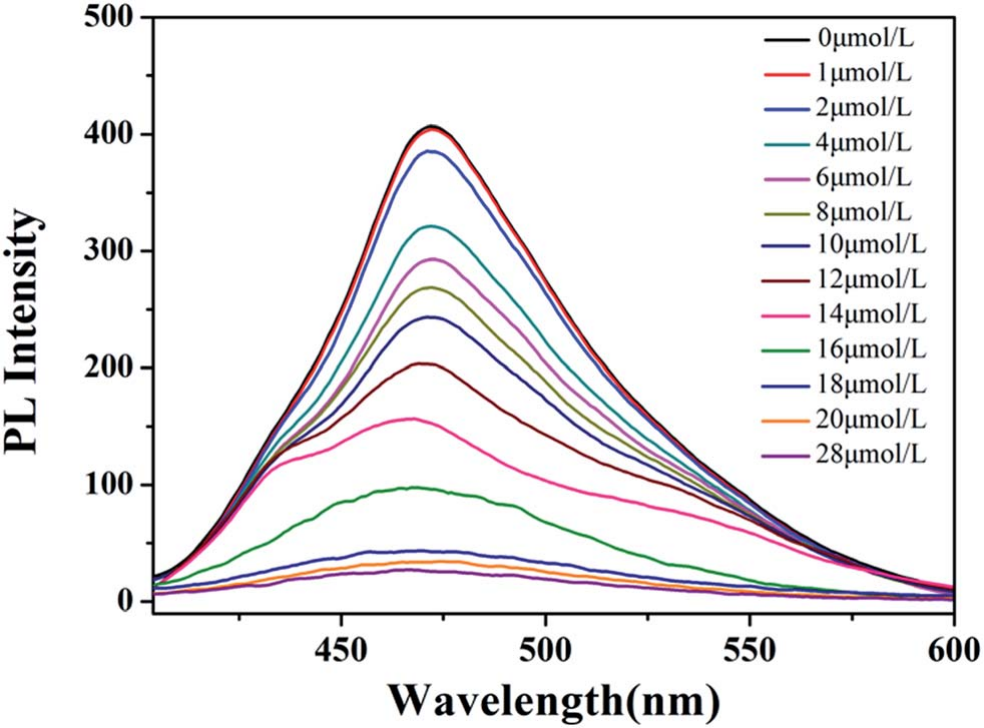
The curves of PL Intensity *vs.* [Zn^2+^] of HPEAM-TPEAH. Polymer concentration: 0.125 mg mL^−1^; test temperature: 25 °C; *λ*_ex_ = 310 nm.

**Fig. 8:**
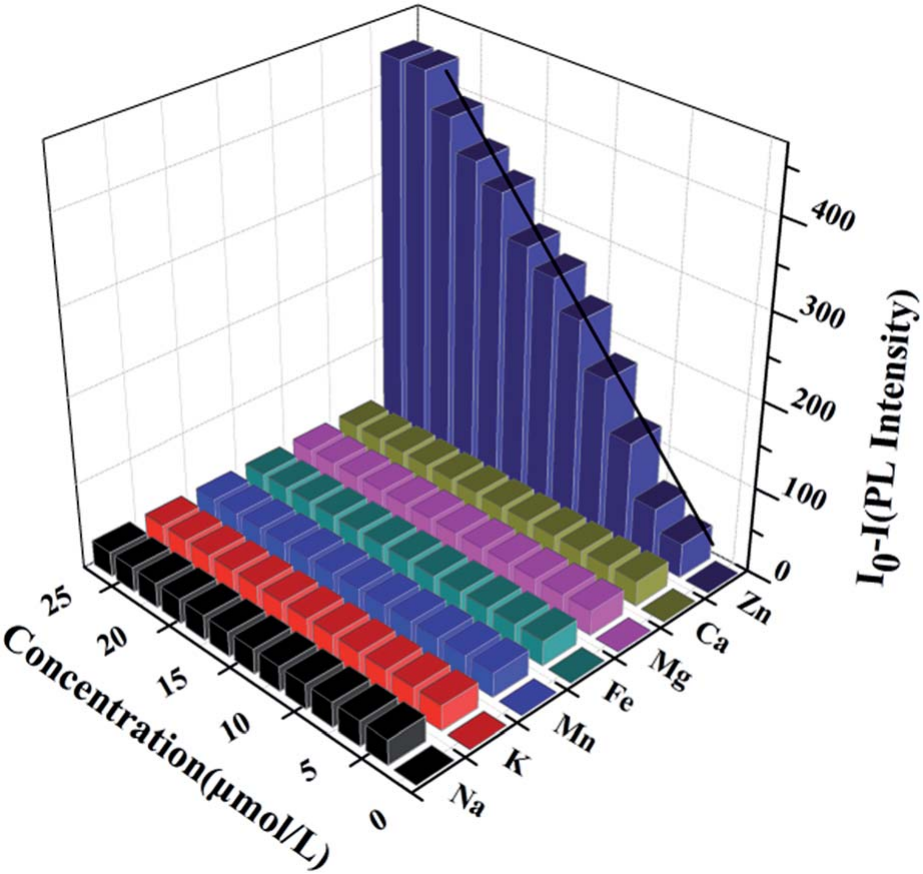
The three-dimensional histogram of *I*_0_–*I vs.* the concentration of metals, which *I*_0_ represents the initial PL intensity, and *I* is the PL intensity under the different concentration of metals. Polymer concentration: 0.125 mg mL^−1^; test temperature: 25 °C.

As aforementioned result shows that the *N*_Zn^2+^_ or the concentration of Zn^2+^ ([Zn^2+^]) can affect the LCST of HPEAM-MBAs, which means [Zn^2+^] can tune the confirmation of the HPEAM-MBAs. Therefore, the [Zn^2+^] should be associated with the PL intensity of HPEAM-TPEAH. As shown in Fig. 7, along with the increase of [Zn^2+^], the PL intensity gradually decrease. When the [Zn^2+^] reaches 0.02 mmol L^−1^, the PL intensity is very low and keeps unchanged even if the [Zn^2+^] is higher. The relationship of PL intensity and concentration of other metals was also investigated and the corresponding curves are shown in ESI.† The PL intensity of HPEAM-TPEAH solution still unchanged, even if the [metals], including Na^+^, K^+^, Ca^2+^, Mg^2+^ and Fe^2+^, reaching 100 mmol L^−1^. The accuracy of the detection of Zn^2+^ by using HPEAM-TPEAH is also evaluated. The plots of [Zn^2+^] against *I*_0_–*I* are displayed in Fig. 8, where *I*_0_ represents the initial PL intensity, and *I* is the PL intensity under the corresponding [Zn^2+^]. Results of the linear regression for *I*_0_–*I* and [Zn^2+^] show that the linear correlation coefficient (*R*^2^) is greater than 0.995 which indicates that the *I*_0_–*I* and [Zn^2+^] shows a good linearity relationship in the range from 4 × 10^−6^ to 1.8 × 10^−5^ mol L^−1^. Traditionally, the synthesis of polymers bearing the detection capacity upon zinc ions were usually based on the introduction of Zn^2+^ recognition units into the backbones.^15,24,25^ For an example, the results given by the literature indicated that the detection specificity would be significantly disturbed when the concentration of other metal ions, such as Na^+^, K^+^, Mg^2+^ and Ca^2+^, reaches 50 mM.^24,25^ In this study, HPEAM-TPEAH was prepared and used for the detection of Zn^2+^ based on the interaction between Zn^2+^ and N and O atoms accompanying with the aggregation-induced emission (AIE) effect. The results indicate that HPEAM-TPEAH holds a desirable specificity on the detection of Zn^2+^ when the concentration of those metal ions reaches 100 mM, where the *I*_0_–*I* and [Zn^2+^] shows a good linearity relationship in the range from 2 × 10^−6^ to 2 × 10^−5^ mol L^−1^.

**Fig. 9:**
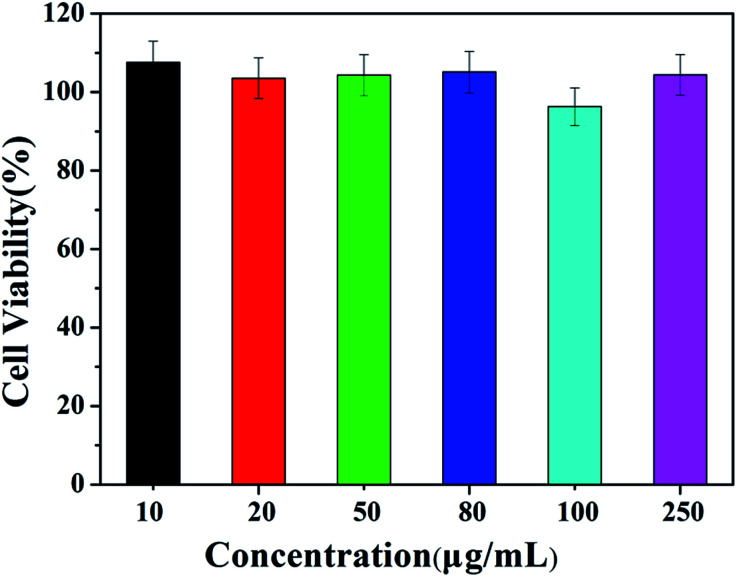
Cell viability of HPEAM-TPEAH at different concentrations.

The cytotoxicity of HPEAM-TPEAH was tested by using Hela cells and the results are given in Fig. 9. HPEAM-TPEAH shows very low cytotoxicity (over 95% cell viability) within 24 h of incubation time, in which the concentration ranges from 1.0 × 10^−6^ to 5.0 × 10^−5^ mol L^−1^.

## Conclusions

In summary, a new thermo-responsive hyperbranched copoly(bis(*N*,*N*-ethyl acryl amide)/(*N*,*N*-methylene bisacrylamide)) (HPEAM-MBA) was synthesized by using RAFT. Zn^2+^ was found to be able to bind with N atom and O atom in the backbone of HPEAM-MBA. When the environment temperature is 20 degrees above the LCST, HPEAM-MBAs solution exhibit high sensitivity in assessing Zn^2+^, which make it possible to gain a new selectively recognizing system. Furthermore, a new Zn^2+^ specific fluorescent probe (HPEAM-TPEAH) was developed by the copolymerization of *N*,*N*-ethyl acryl amide and TPEAH in this study. The introduction of TPE units gives a highly specific and sensitive Zn^2+^-sensitivity to HPEAM-TPEAH, as a result the fluorescence exhibits a highly specific “turn-off” response for Zn^2+^.

## Conflicts of interest

There are no conflicts to declare.

## Supplementary Material

RA-008-C7RA13263H-s001
