# Supplementary material for: Facile construction of a hyperbranched poly(acrylamide) bearing tetraphenylethene units: a novel fluorescence probe with a highly selective and sensitive response to Zn2+
Source: RSC Adv. 2018 Feb 5;8(11):5776–83. doi: 10.1039/c7ra13263h (PMC9078268; doi:10.1039/c7ra13263h)
Supplement: RA-008-C7RA13263H-s001 [file RA-008-C7RA13263H-s001.pdf]

Electronic Supplementary Material (ESI) for RSC Advances.
This journal is © The Royal Society of Chemistry 2018

Facile Construction of a Hyperbranched Polyamine Bearing Tetraphenylethene Units: A Novel Fluorescence Probe with Highly Selective and Sensitive Response to Zn^{2+}

----Supporting Information

Xuejing Liu[a], Yuangong Zhang[a], Haijing Hao[a], Wanju Zhang[b], Libin Bai[a,b], Yonggang Wu[a], Hongchi Zhao[a], Hailei Zhang[a*] and Xinwu Ba[a]*

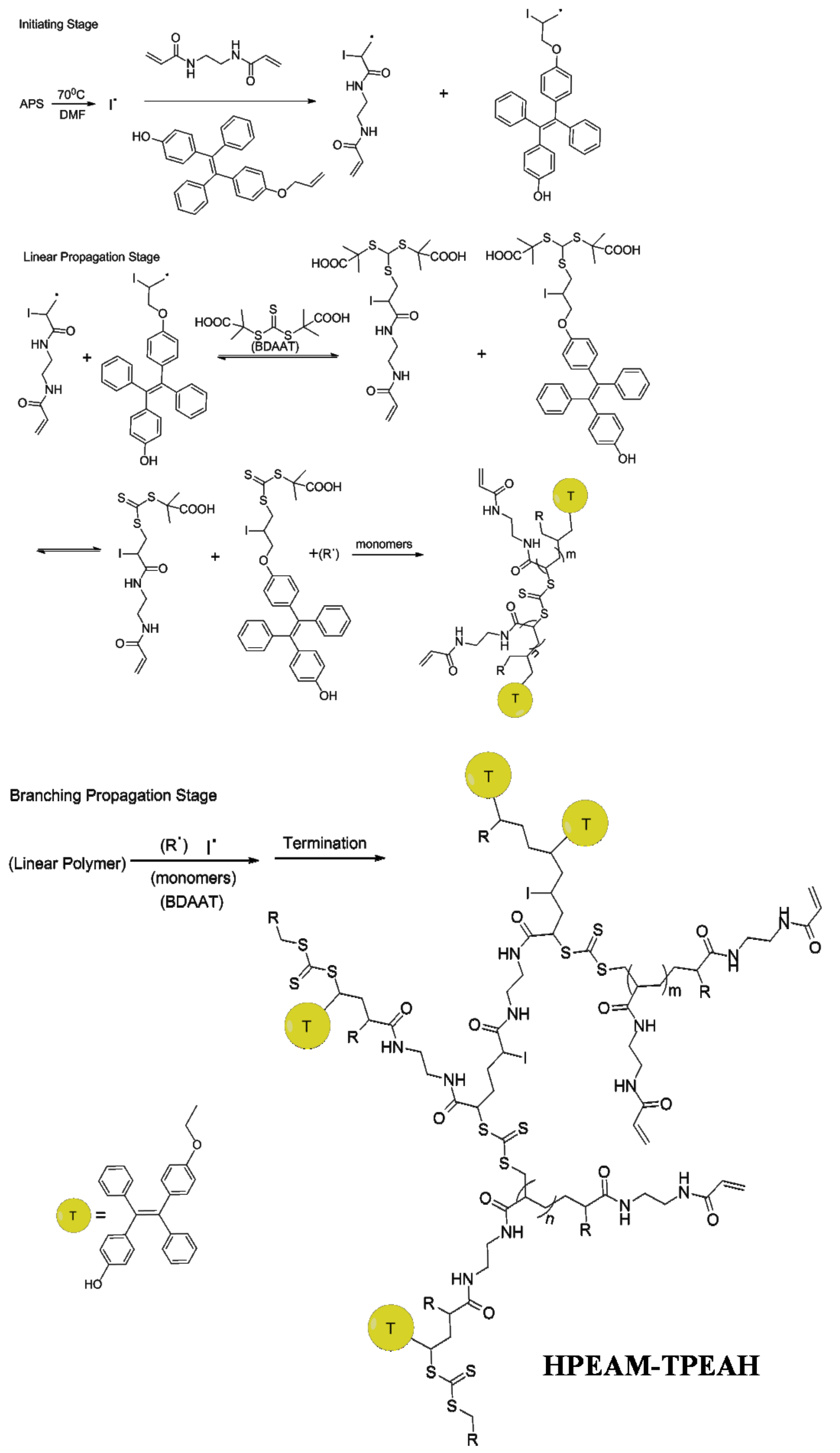

Initiating Stage
APS
70⁰C
DMF
I•
Linear Propagation Stage
(BDAAT)
monomers
+(R')
Branching Propagation Stage
(Linear Polymer)
(R') I•
(monomers)
(BDAAT)
Termination
HPEAM-TPEAH

Scheme S1. The synthesis of HPEAM-TPE: In the initiating stage, the initiator APS is decomposed to initial free radical under 70 °C, then the monomers are initiated by the initial free radicals that are consistent with the established mechanism of RAFT polymerization. The second stage is linear propagation stage. In the branching propagation stage, the vinyl groups as the side groups possess reaction activity and can be initiated by the active species. Therefore, the branched structure will be increased with the conversion of the vinyl groups. For the multi vinyl monomer, it will be polymerized, forming a crosslinked polymer rather than a hyperbranched polymer due to the high reactivity of vinyl groups. Thus, RAFT has to be occupied for slowing down the growth rate and avoiding rapid cross-linking. Finally, the HPEAM-TPEAM is obtained bearing amount of vinyl groups and thioester.

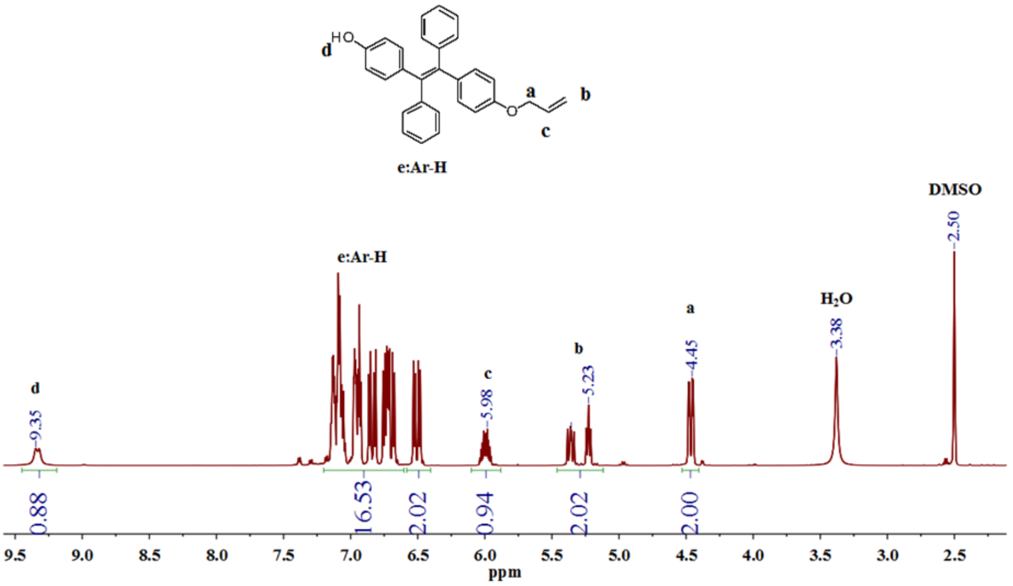

Figure S1. ^{1}H NMR (CD_3SOCD_3) spectrum of TPEAH.

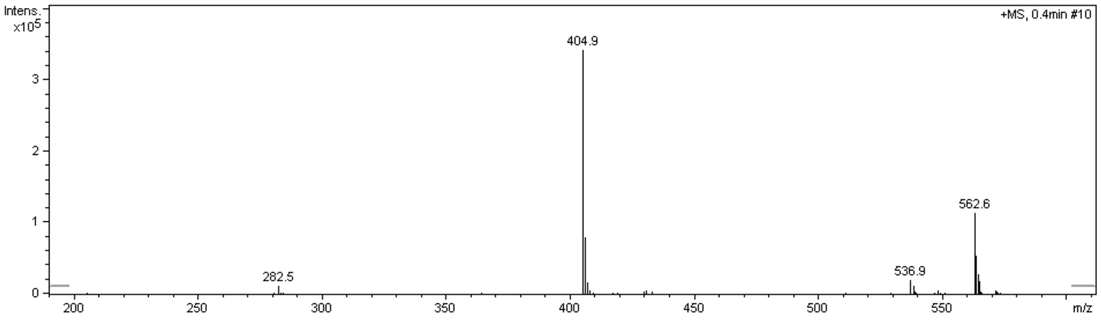

Figure S2. MS spectrum of TPEAH.

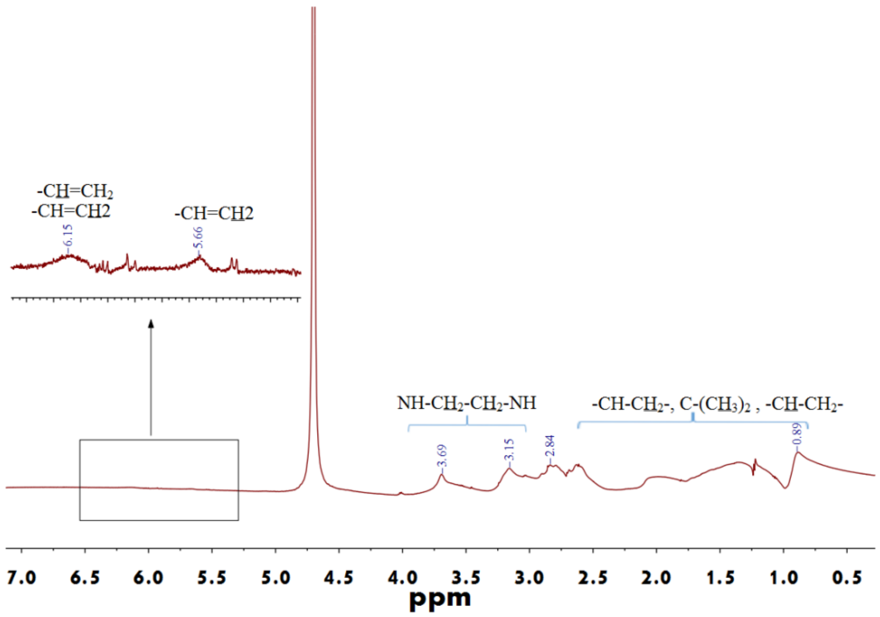

Figure S3. ^{1}H NMR (D_2O) spectrum of HPEAM-MBA.

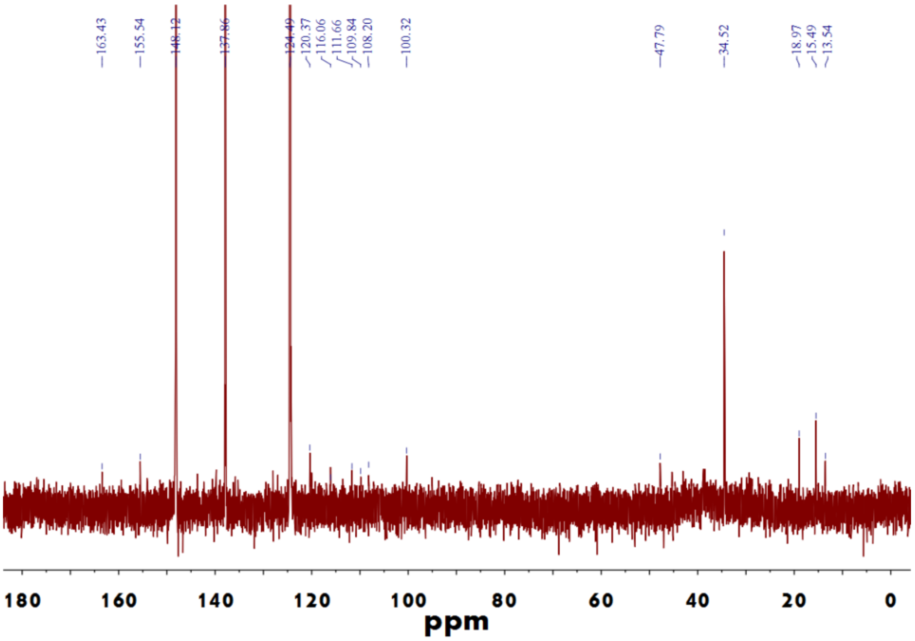

Figure S4. ^{13}C NMR(D_2O) spectrum of HPEAM-MBA.

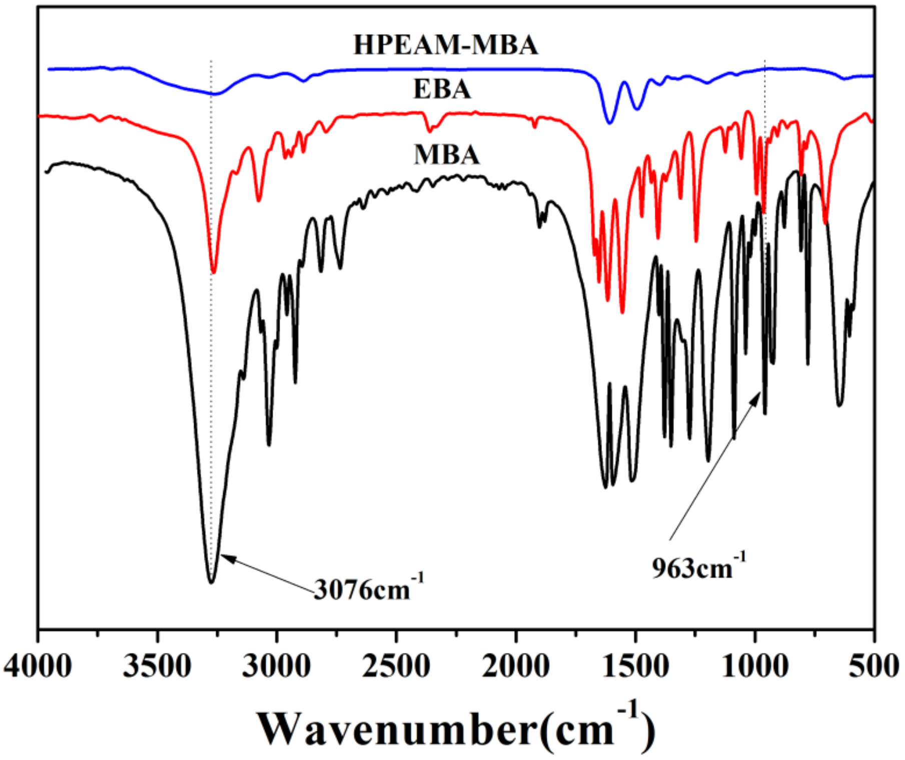

Figure S5. FT-IR spectrum of HPEAM-MBA.

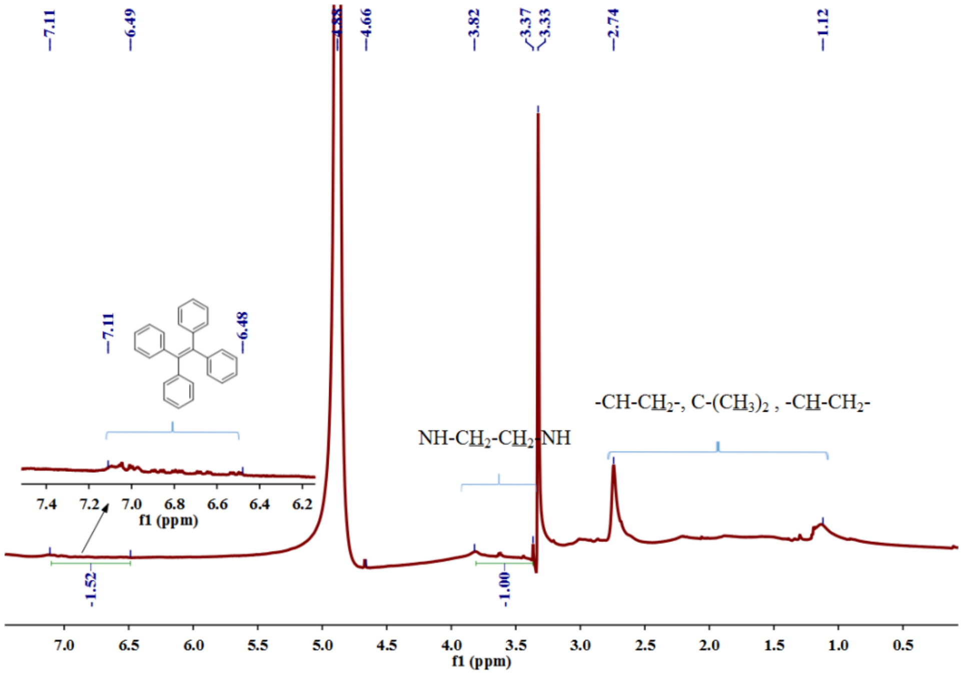

Figure S6. ^{1}H NMR (CD_3OD) spectrum of HPEAM-TPEAH.

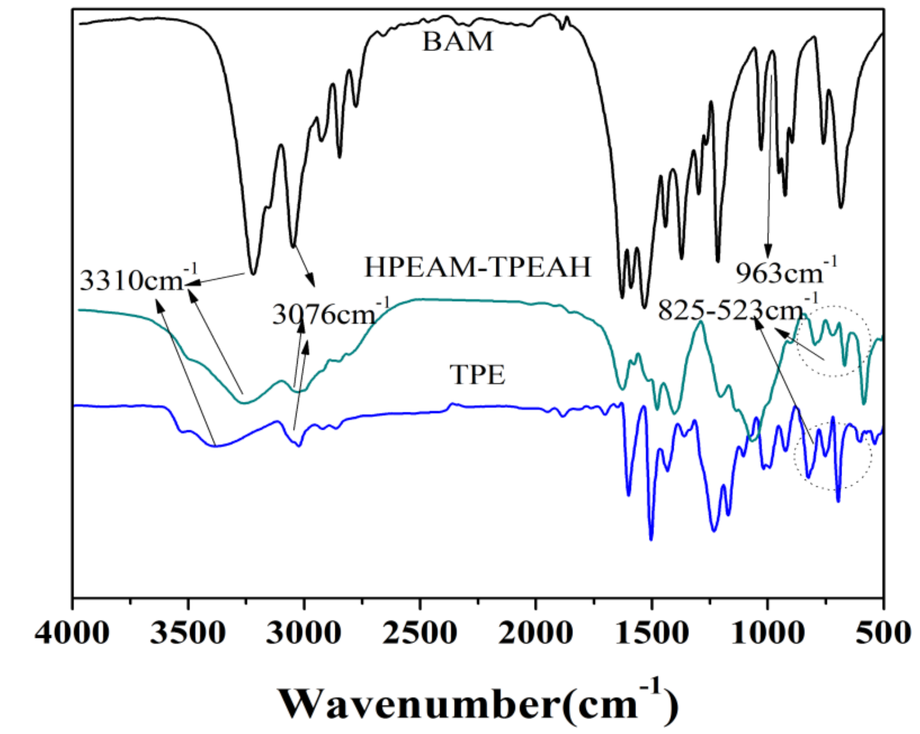

Figure S7. FT-IR spectrum of HPEAM-TPEAH.

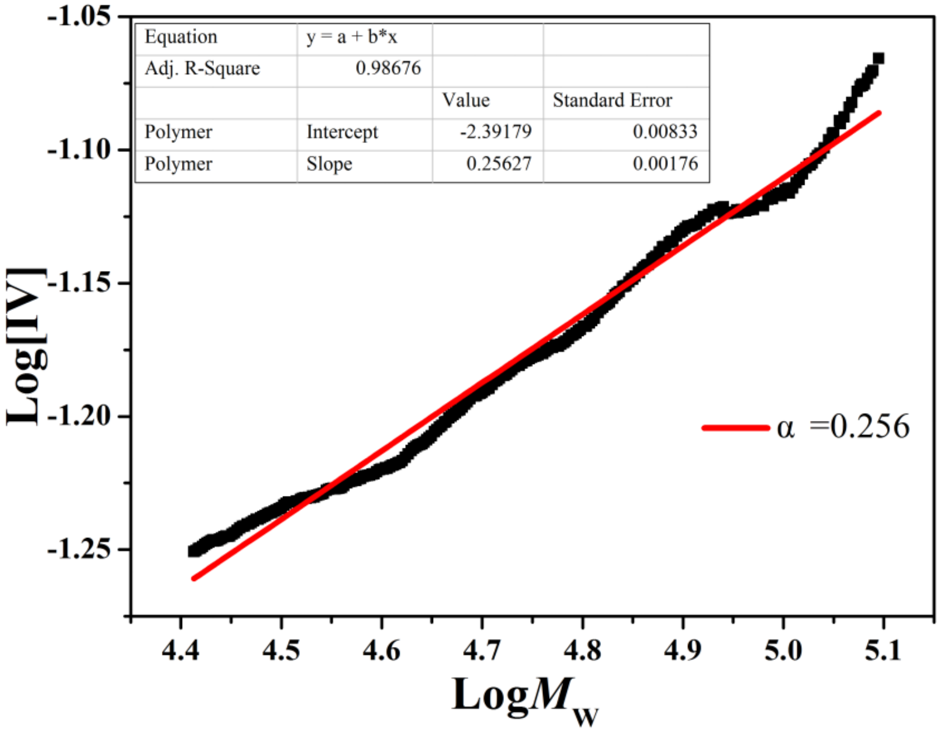

Figure S8. The plots of Log M_w vs. Log η

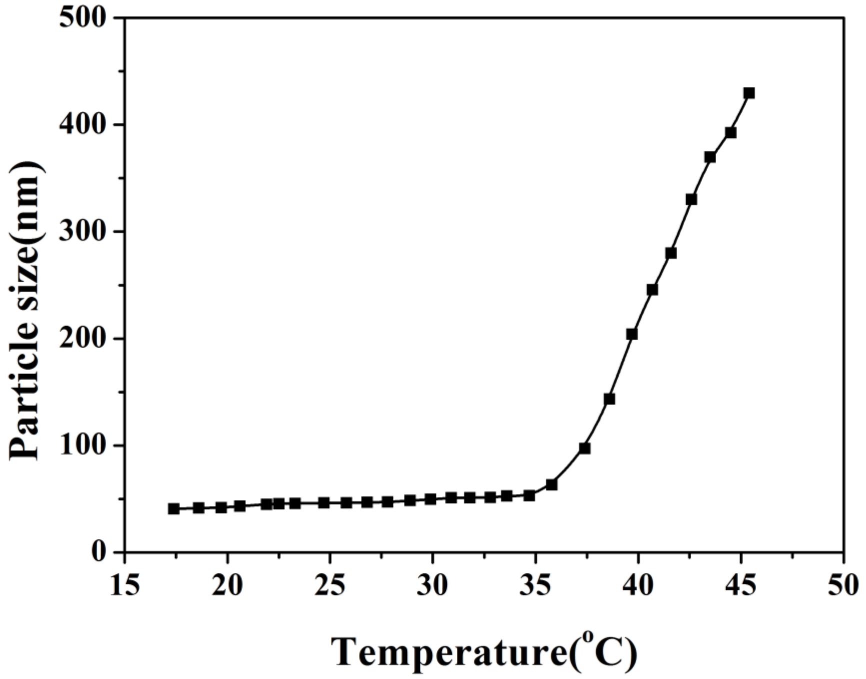

Figure S9. The diameter of nanomicell vs. temperature for each of the temperature, measuring the diameter after holding the temperature 10 min. Solution concentration of HPEAM-MBA: 1 mg mL^{-1}; heating rate: 0.1 ^{o}C min^{-1}; pH=7.

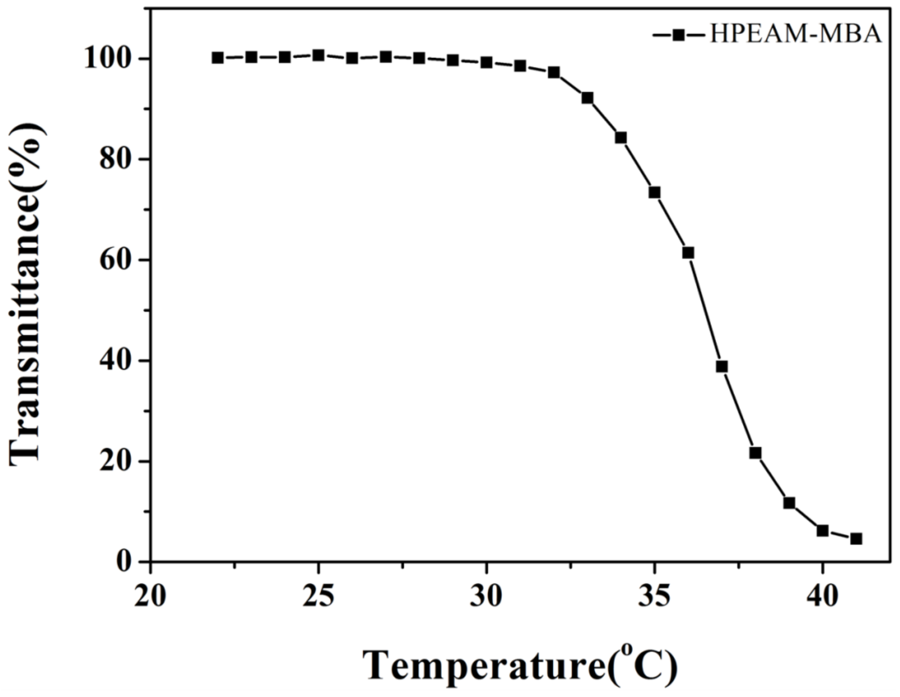

Figure S10. The curves of transmittance versus temperature of HPEAM-MBA solution. Polymer concentration: 5 mg mL^{-1}; heating rate: 0.1 ^{o}C min^{-1}.

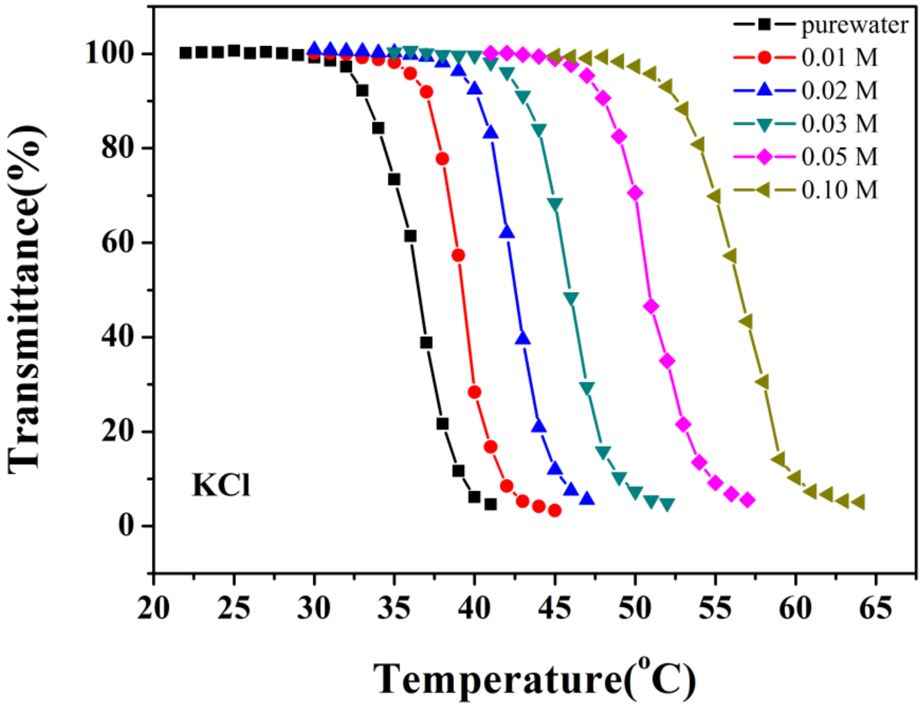

Figure S11. The curves of transmittance versus temperature of HPEAM-MBA solution containing K^+. Polymer concentration: 5 mg mL^{-1}; heating rate: 0.1 ^oC min^{-1}.

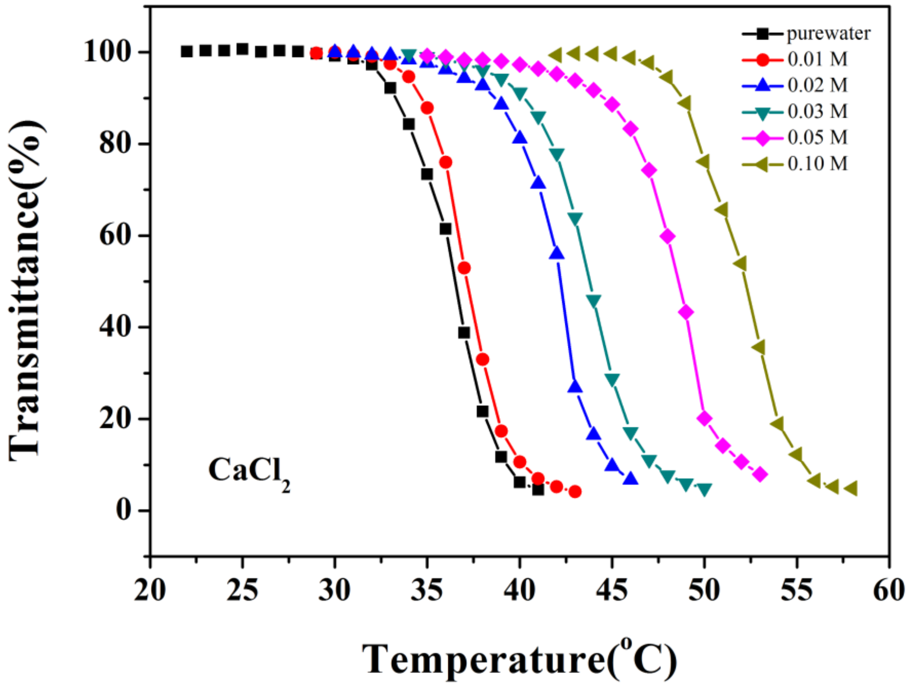

Figure S12. The curves of transmittance versus temperature of HPEAM-MBA solution containing Ca^{2+}. Polymer concentration: 5 mg mL^{-1}; heating rate: 0.1 ^oC min^{-1}.

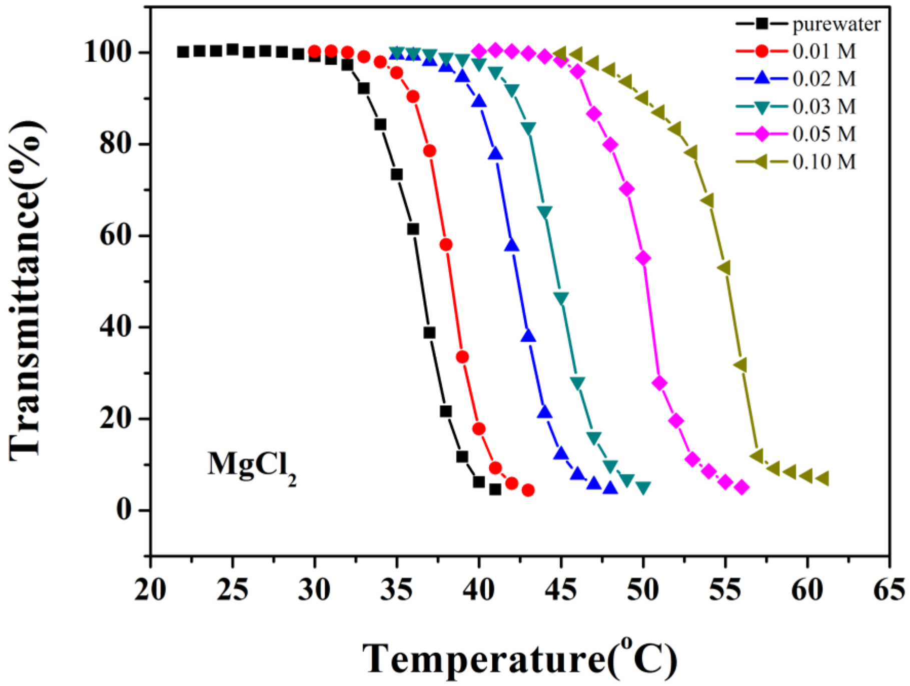

Figure S13. The curves of transmittance versus temperature of HPEAM-MBA solution containing Mg^{2+}. Polymer concentration: 5 mg mL^{-1}; heating rate: 0.1 °C min^{-1}.

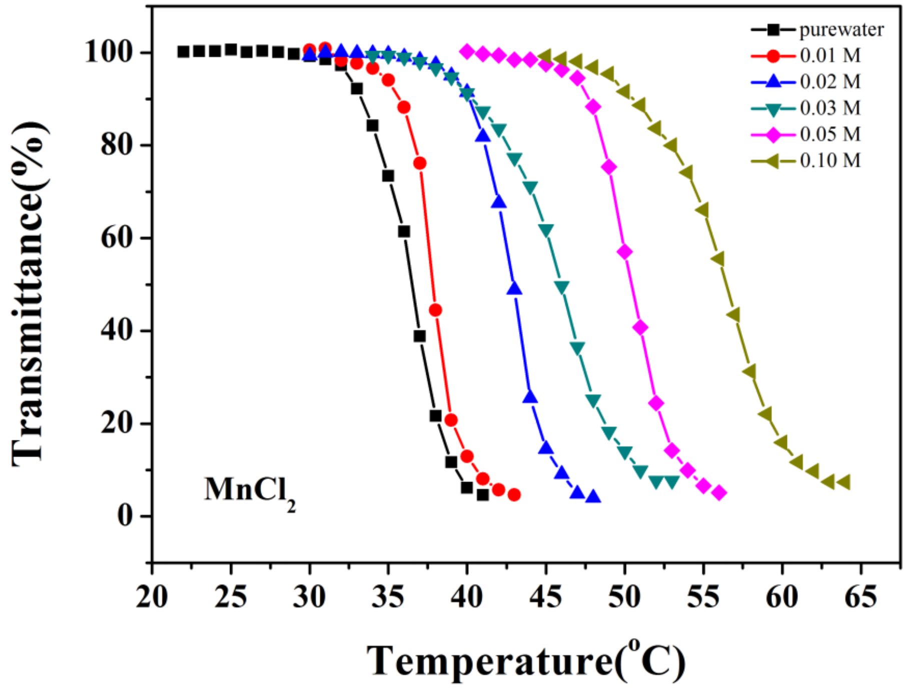

Figure S14. The curves of transmittance versus temperature of HPEAM-MBA solution containing Mn^{2+}. Polymer concentration: 5 mg mL^{-1}; heating rate: 0.1 °C min^{-1}.

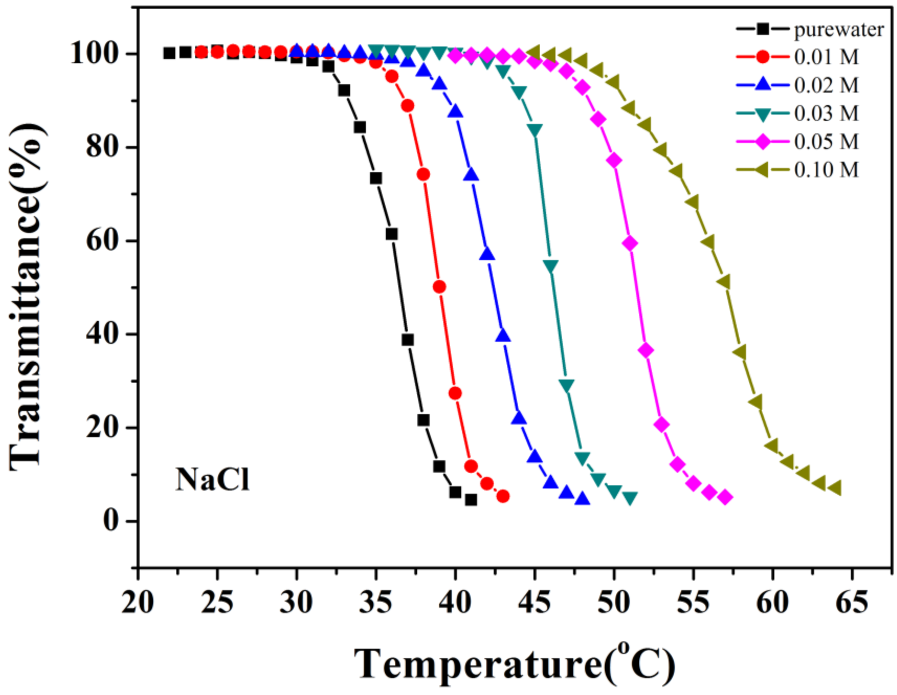

Figure S15. The curves of transmittance versus temperature of HPEAM-MBA solution containing Na^{+}. Polymer concentration: 5 mg mL^{-1}; heating rate: 0.1 °C min^{-1}.

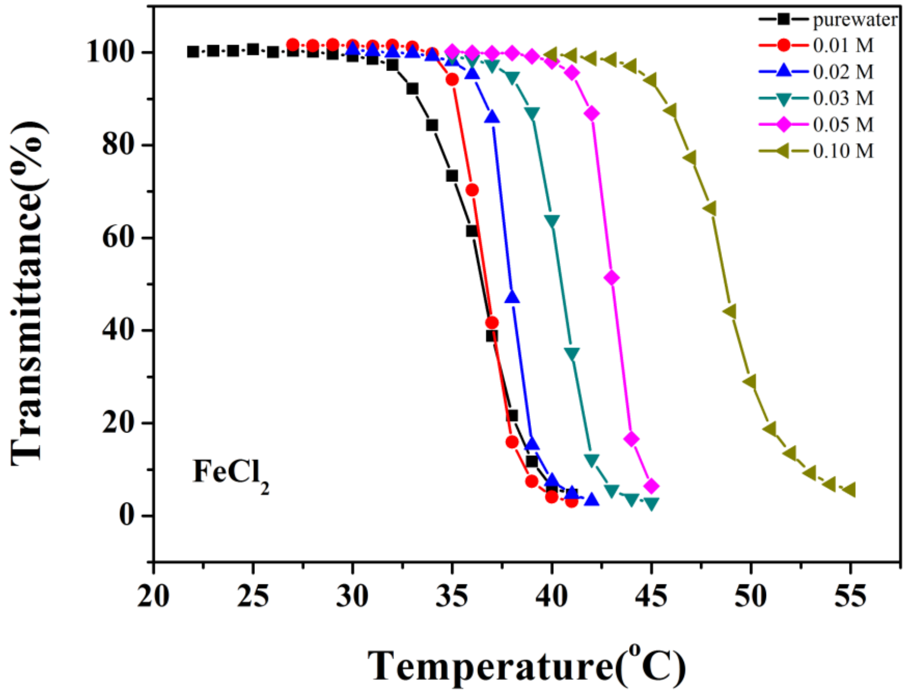

Figure S16. The curves of transmittance versus temperature of HPEAM-MBA solution containing Fe^{2+}. Polymer concentration: 5 mg mL^{-1}; heating rate: 0.1 °C min^{-1}.

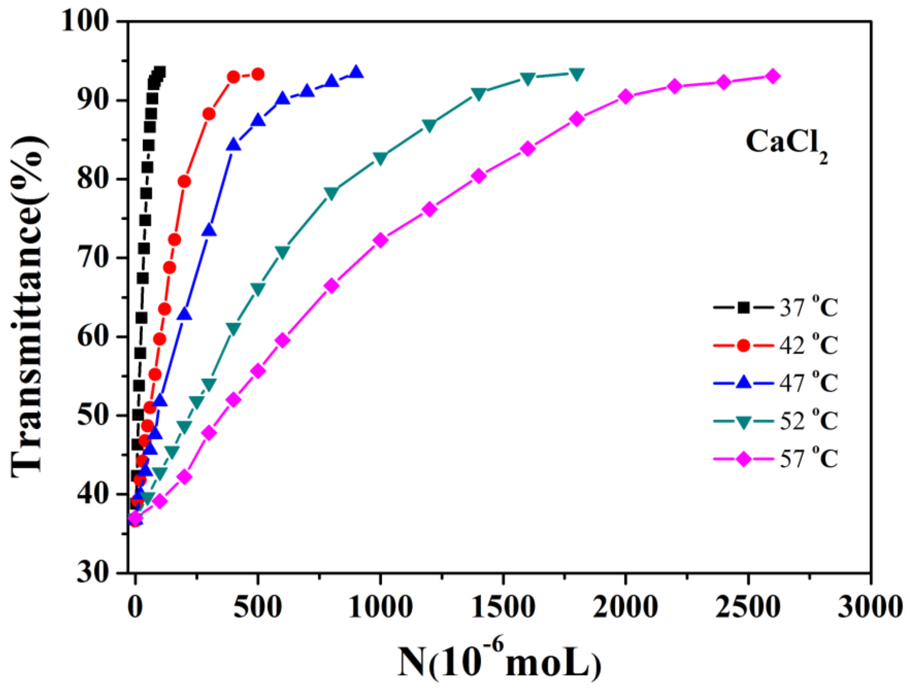

Figure S17. The curves of transmittance% vs. N_{Ca} of HPEAM-MBA at different temperatures. Polymer concentration: 5 mg mL^{-1}.

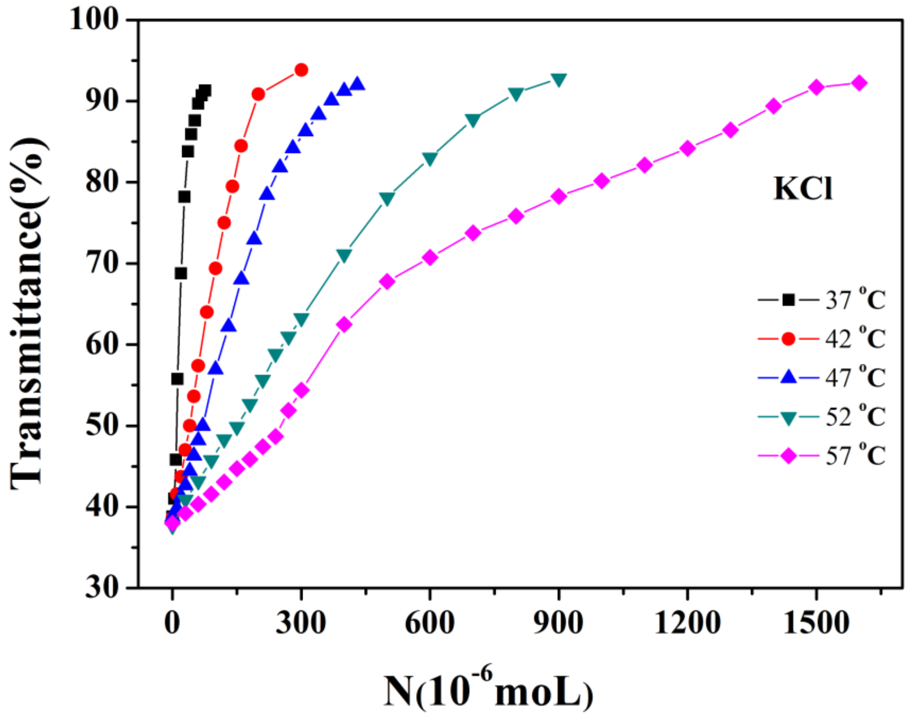

Figure S18. The curves of transmittance% vs. N_K of HPEAM-MBA at different temperatures. Polymer concentration: 5 mg mL^{-1}.

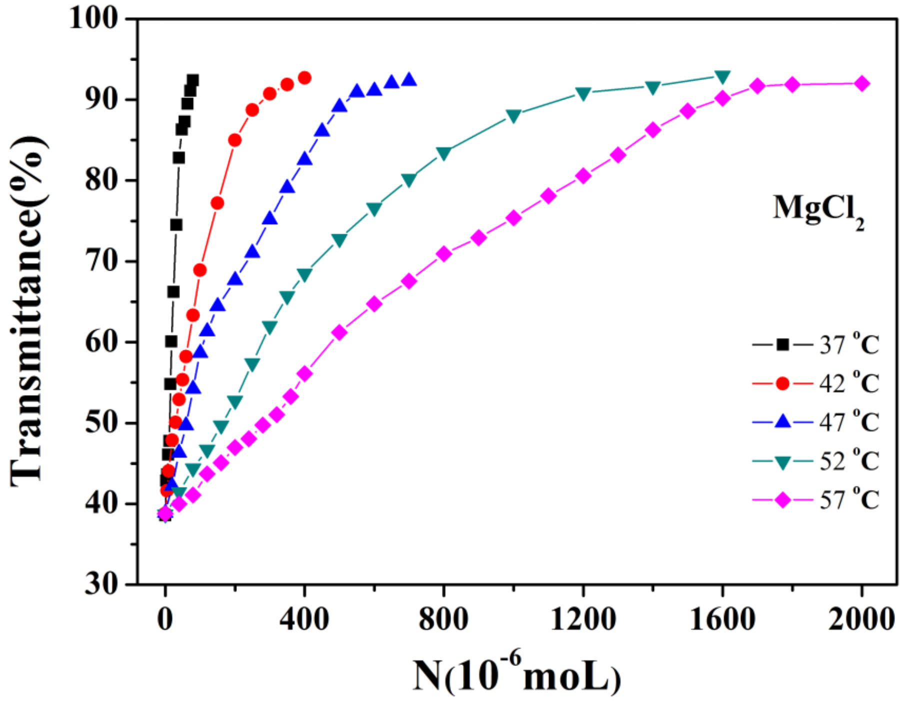

Figure S19. The curves of transmittance% vs. N_{Mg} of HPEAM-MBA at different temperatures. Polymer concentration: 5 mg mL^{-1}.

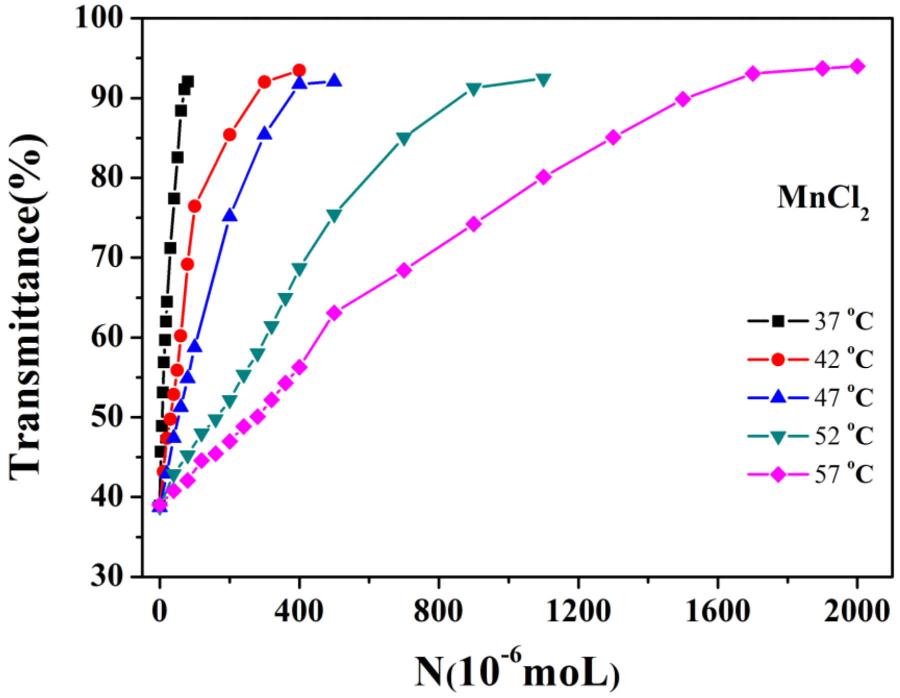

Figure S20. The curves of transmittance% vs. N_{Mn} of HPEAM-MBA at different temperatures. Polymer concentration: 5 mg mL^{-1}.

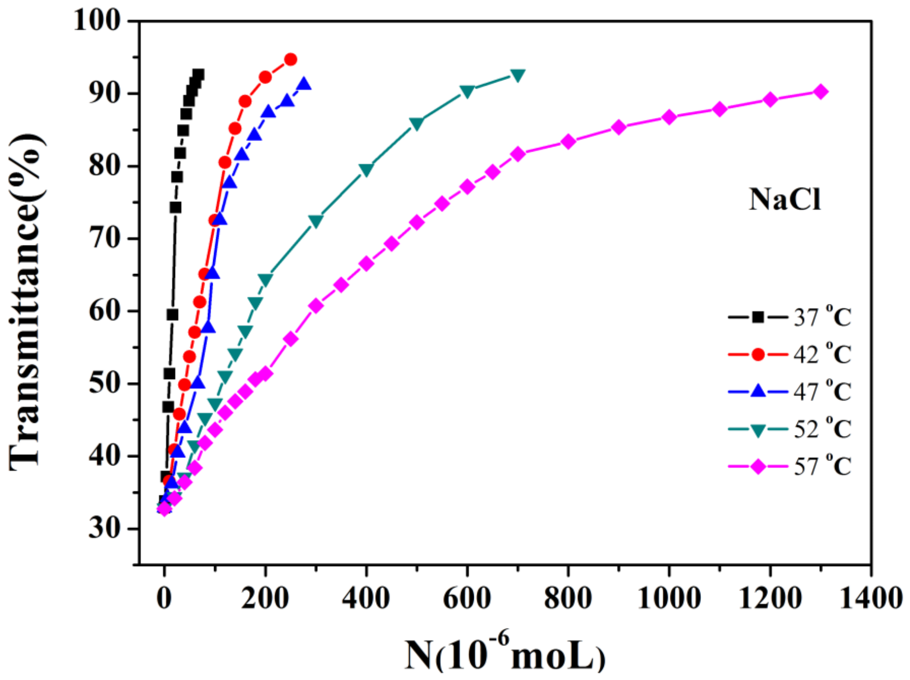

Figure S21. The curves of transmittance% vs. N_{Na} of HPEAM-MBA at different temperatures. Polymer concentration: 5 mg mL^{-1}.

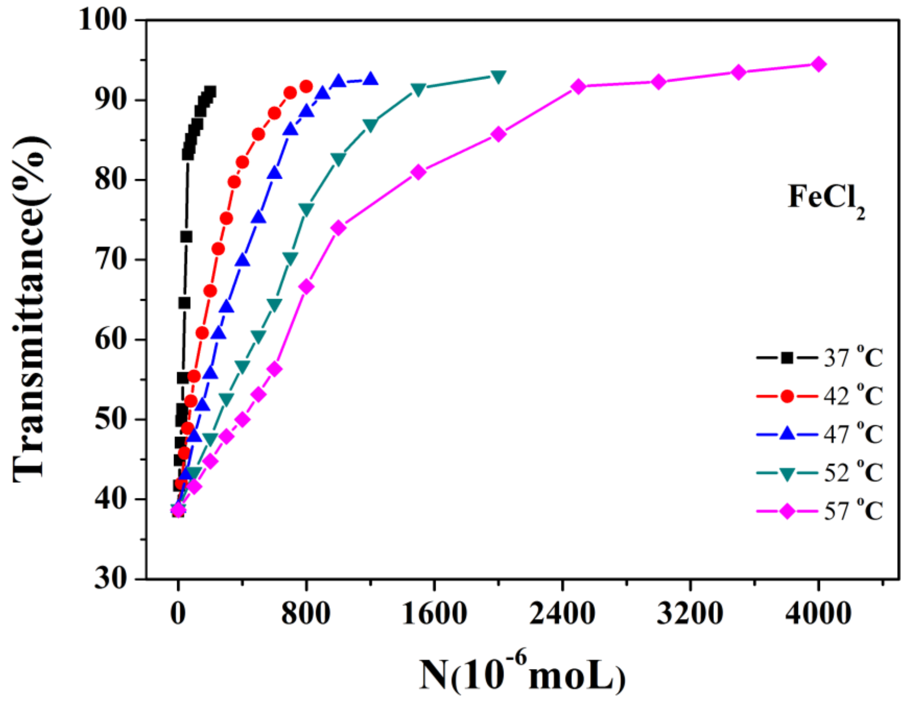

Figure S22. The curves of transmittance% vs. N_{Fe} of HPEAM-MBA at different temperatures. Polymer concentration: 5 mg mL^{-1}.

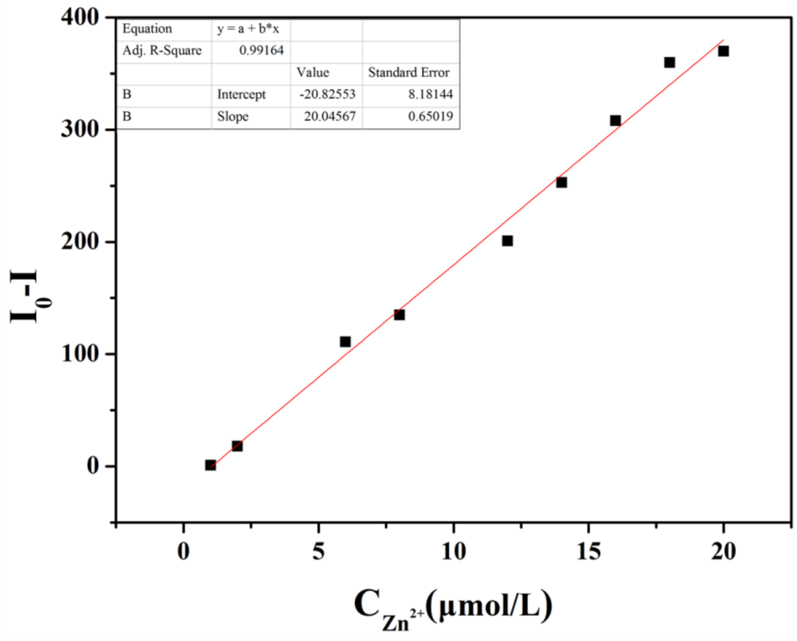

Figure S23. I_0-I vs. [Zn^{2+}] of HPEAM-TPEAH in presence of other interfering ions. The linear correlation coefficient (R^2) is greater than 0.99 which indicates that the I_0-I and [Zn^{2+}] shows a good linearity relationship in the range from 2×10^{-6} to 2×10^{-5} mol/L.

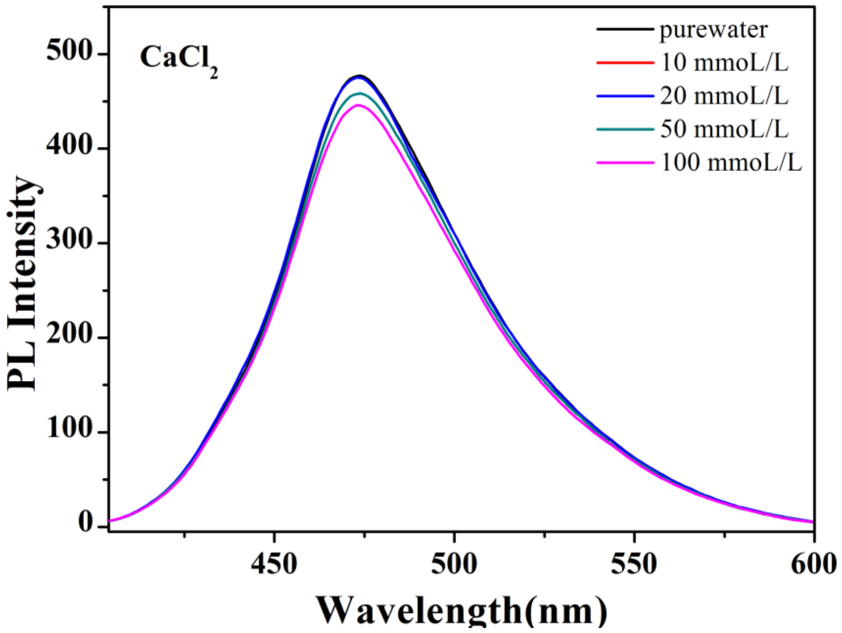

Figure S24. The curves of PL Intensity of HPEAM-TPEAH at different [Ca^{2+}]. Polymer concentration: 0.125 mg mL^{-1}; test temperature: 25 ºC; λ_{ex}=310 nm.

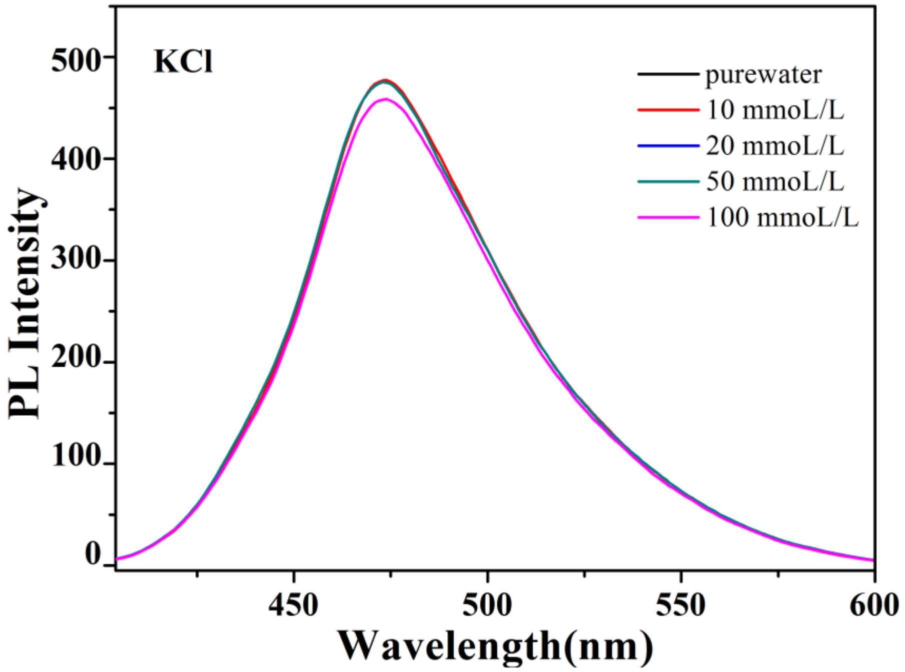

Figure S25. The curves of PL Intensity of HPEAM-TPEAH at different [K^+]. Polymer concentration: 0.125 mg mL^{-1}; test temperature: 25 ºC; λ_{ex}=310 nm.

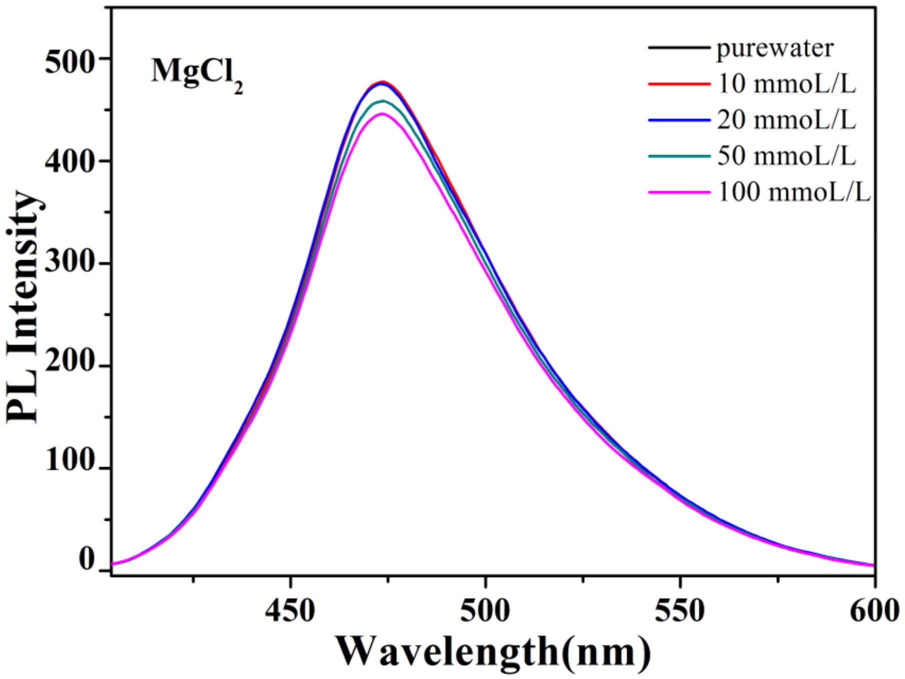

Figure S26. The curves of PL Intensity of HPEAM-TPEAH at different [Mg^{2+}]. Polymer concentration: 0.125 mg mL^{-1}; test temperature: 25 ºC; λ_{ex}=310 nm.

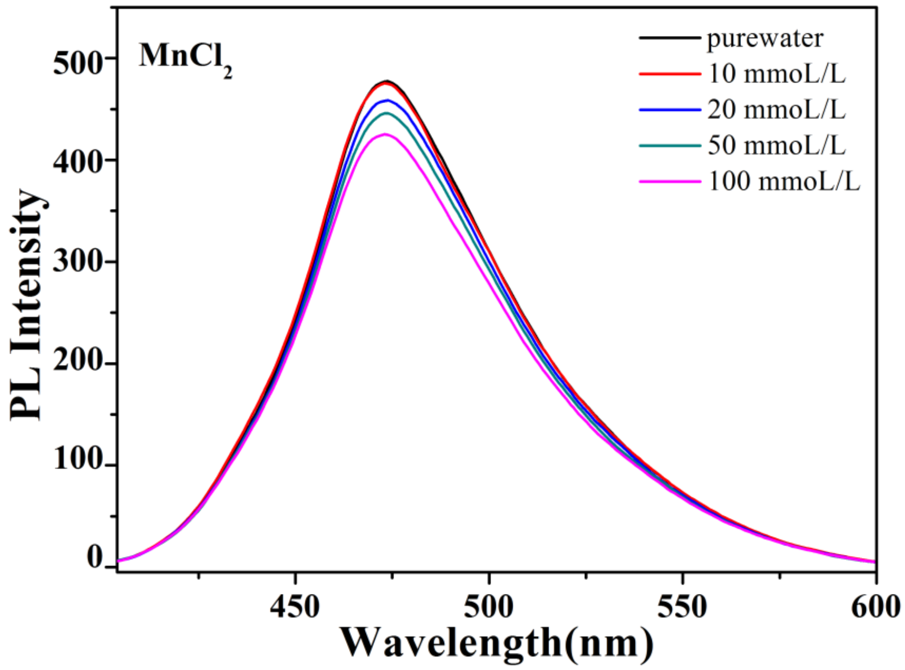

Figure S27. The curves of PL Intensity of HPEAM-TPEAH at different [Mn^{2+}]. Polymer concentration: 0.125 mg mL^{-1}; test temperature: 25 ºC; λ_{ex}=310 nm.

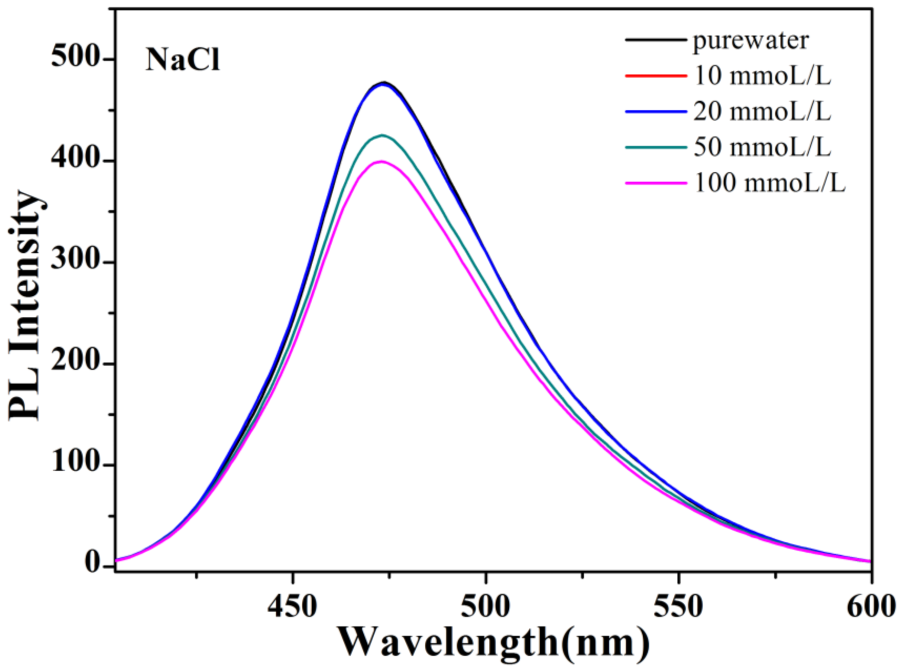

Figure S28. The curves of PL Intensity of HPEAM-TPEAH at different [Na^{+}]. Polymer concentration: 0.125 mg mL^{-1}; test temperature: 25 ºC; λ_{ex}=310 nm.

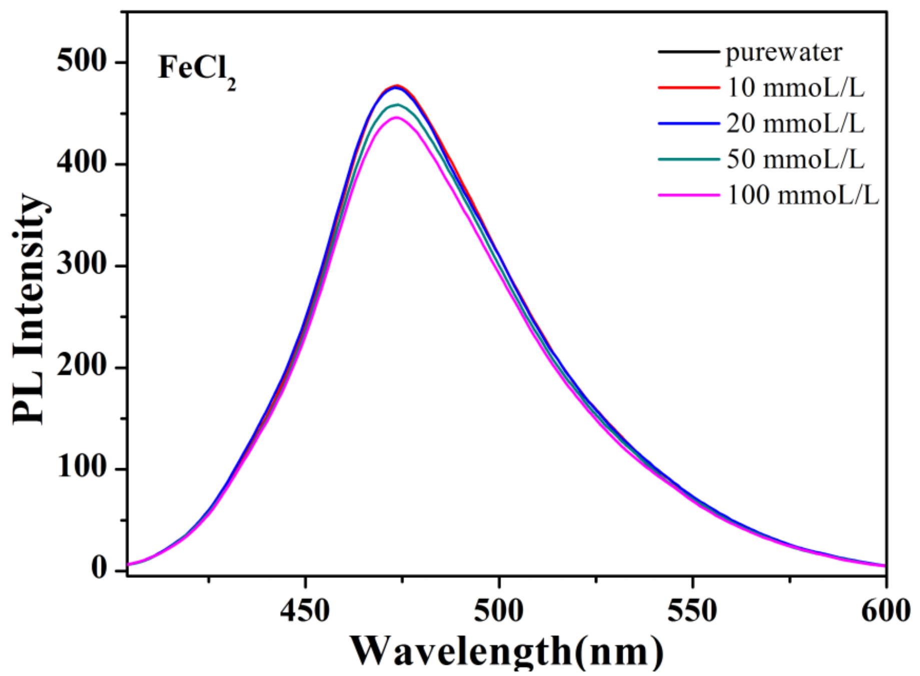

Figure S29. The curves of PL Intensity of HPEAM-TPEAH at different [Fe^{2+}]. Polymer concentration: 0.125 mg mL^{-1}; test temperature: 25 °C; λ_{ex}=310 nm.
